# Task Instructions and the Need for Feedback Correction Influence the Contribution of Visual Errors to Reach Adaptation

**DOI:** 10.1523/ENEURO.0068-23.2023

**Published:** 2023-09-04

**Authors:** Anne H. Hoffmann, Frédéric Crevecoeur

**Affiliations:** 1 Institute for Information and Communication Technologies, Electronics and Applied Mathematics (ICTEAM), Université Catholique de Louvain, Louvain-la-Neuve 1348, Belgium; 2 Institute of Neuroscience (IoNS), Université Catholique de Louvain, Brussels 1200, Belgium

**Keywords:** motor adaptation, motor control, multisensory integration, proprioception, reaching movements, vision

## Abstract

Previous research has questioned whether motor adaptation is shaped by an optimal combination of multisensory error signals. Here, we expanded on this work by investigating how the use of visual and somatosensory error signals during online correction influences single-trial adaptation. To this end, we exposed participants to a random sequence of force-field perturbations and recorded their corrective responses as well as the after-effects exhibited during the subsequent unperturbed movement. In addition to the force perturbation, we artificially decreased or increased visual errors by multiplying hand deviations by a gain smaller or larger than one. Corrective responses to the force perturbation clearly scaled with the size of the visual error, but this scaling did not transfer one-to-one to motor adaptation and we observed no consistent interaction between limb and visual errors on adaptation. However, reducing visual errors during perturbation led to a small reduction of after-effects and this residual influence of visual feedback was eliminated when we instructed participants to control their hidden hand instead of the visual hand cursor. Taken together, our results demonstrate that task instructions and the need to correct for errors during perturbation are important factors to consider if we want to understand how the sensorimotor system uses and combines multimodal error signals to adapt movements.

## Significance Statement

We investigated the factors influencing visual and proprioceptive feedback contributions to movement control and adaptation. While online corrections increased with the size of visual errors, this scaling did not transfer one-to-one to adaptation. Instead, we observed a consistent relationship between limb displacement during perturbation and subsequent after-effects which was independent of the visual error. However, adaptation was slightly reduced when visual errors were artificially decreased and this influence of vision was modulated by the task-instruction given to participants. Our results demonstrate that task-related factors, such as the need to correct movement errors and the instructions, have to be considered to advance our understanding of how the sensorimotor system uses multisensory feedback to adapt motor commands.

## Introduction

When we reach for a glass of water, visual feedback about the position of the glass and the hand needs to be combined with proprioceptive information about the hand location originating from somatosensory afferents. If the environment is static, the brain is able to combine different sensory cues in a near optimal manner to minimize estimation uncertainty (van Beers et al., 1999; Ernst and Banks, 2002). However, during motion, the feedback the brain receives changes as a function of time. This complicates the task of integrating visual and proprioceptive signals while maintaining stable movement control because each sensory modality possesses a different processing delay (Cluff et al., 2015; Oostwoud Wijdenes and Medendorp, 2017). Recent studies have proposed that the brain may be able to solve this challenge by accounting for differences in feedback delays and dynamics when integrating vision and proprioception (Crevecoeur et al., 2016; Kasuga et al., 2022).

Besides responding to immediate sensory errors, the brain also learns from past errors to adapt to perturbations or other changes in the environment. Studies on blind or deafferented patients have shown that some degree of movement adaptation can occur even in the absence of visual or proprioceptive feedback (Dizio and Lackner, 2000; Sarlegna et al., 2006, 2010). This shows that the brain can compensate for sensory loss in a highly flexible manner. However, even when vision is intact, some studies have reported that visual feedback is not necessary or plays a reduced role during adaptation (Bernier et al., 2005; Franklin et al., 2007; Batcho et al., 2016; McKenna et al., 2017). Interestingly, in other contexts it has been observed that vision dominates adaptation and can even override proprioceptive inputs when there is conflict between the two senses (Scheidt et al., 2005; Judkins and Scheidt, 2014). Again, others have reported that vision and proprioception both contribute to adaptation but that the integration of multisensory errors does not follow the predictions of the static optimal cue integration model. In particular, adaptation does not seem to increase linearly with the size of different error signals but instead reaches an asymptote as errors increase (Marko et al., 2012; Hayashi et al., 2020). Importantly, when we compare these seemingly contradictory results, it should be noted that these studies used a large variety of different tasks, perturbations, and instructions. It remains unclear which of these factors might determine whether or how visual and proprioceptive errors contribute to the adaptation of movements.

To address this question, we investigated the influence of combined visual and proprioceptive errors on online corrections and single-trial adaptation using a random force-field adaptation paradigm. A randomized perturbation schedule prevents anticipatory compensation for the force-field disturbances, thus enabling the measurement of corrective responses to movement errors. However, clear after-effects are observed even after a single exposure to a force-field, providing a direct measure of single-trial adaptation (Sing et al., 2013; Crevecoeur et al., 2020; Mathew et al., 2020). In addition to the force-field, we introduced a mismatch between the visual cursor feedback and the actual hand position by multiplying the lateral cursor position by a gain smaller, equal, or larger than one. The visual gain (VG) resulted in a naturalistic divergence between vision and proprioception by keeping the dynamics of the perturbations similar, thus increasing the probability that vision and proprioception are integrated (Marko et al., 2012; Kasuga et al., 2022). We conducted four experiments in which we manipulated different parameters that might influence proprioceptive and visual contributions to adaptation. In addition to manipulating visual error size (experiment 1), we varied the size of the force perturbation to see whether after-effects saturate for larger perturbations (experiment 2). Further, we altered the instruction whether participants had to respond to perturbations or not (experiment 3) and whether they should control the visual cursor or their hidden hand (experiment 4).

When feedback corrections were required to perform the task, we observed a consistent scaling of feedback responses with visual error size. However, the influence of vision on adaptation measured during the subsequent movement did not systematically scale with the size of the visual perturbation. Instead, the size of after-effects depended on the hand displacement during the perturbation as well as on the task instructions. Taken together, our results show that the extent to which the brain uses visual and proprioceptive errors to update motor commands is influenced by contextual factors, such as the task and the need for feedback corrections.

## Materials and Methods

### Participants

A total of 70 healthy human adults aged 19–40 (mean age 24.6) participated in this series of experiments. Fourteen (nine female) volunteers participated in experiment 1, 16 (11 female) in experiment 2, 24 (18 female) in experiment 3, and 16 (seven female) in experiment 4. Human participants were recruited among the student population of Université Catholique de Louvain (UCLouvain). All participants were right-handed, had no reported neurologic disorders, and had normal or corrected to normal vision. Before their participation all volunteers provided written informed consent. The experiments were performed in agreement with the ethical guidelines of UCLouvain and the St. Luc Hospital. As a compensation for their time, all participants received a small financial reimbursement.

### Apparatus and general task procedure

**Figure 1.**
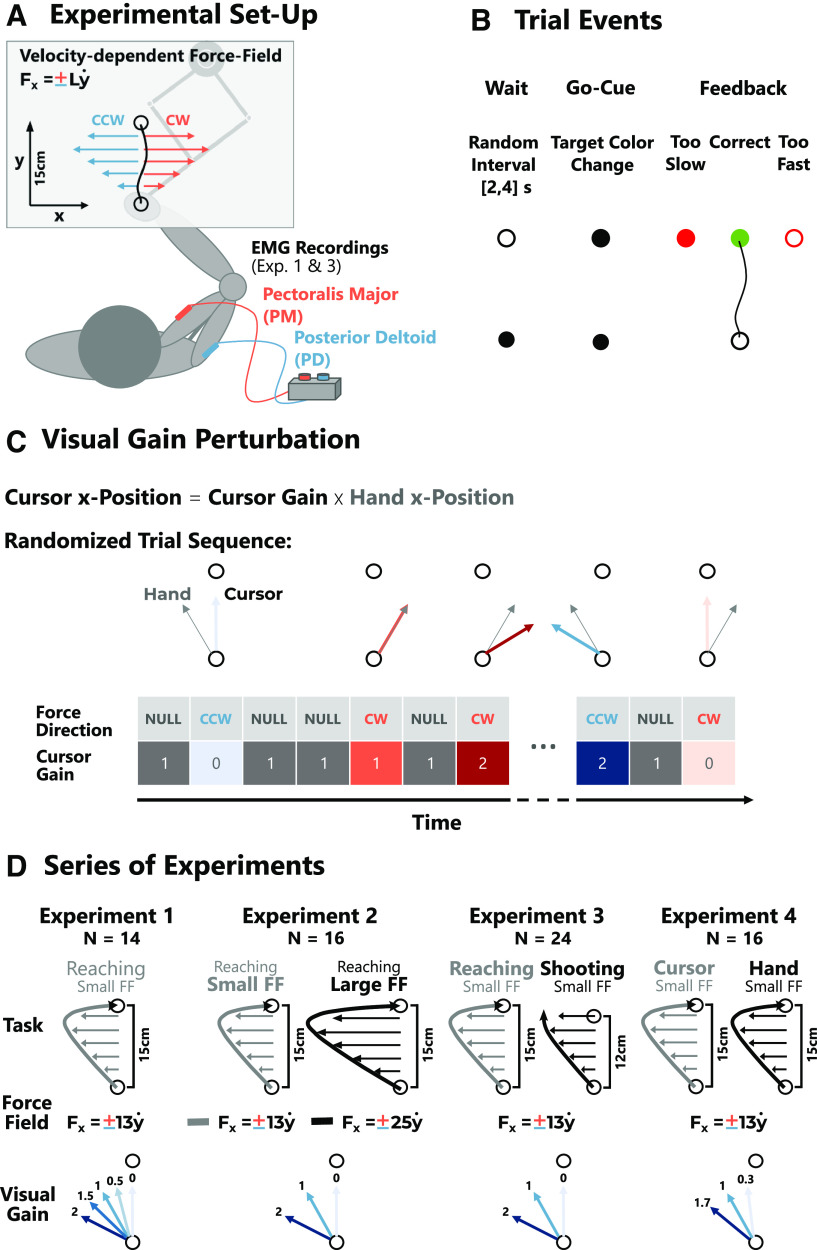
Experimental paradigm. ***A***, Top-view of the work-space showing the orientation of the movements and a sketch of the applied force-field (L = force-field constant) as well as the recorded muscles. ***B***, Depiction of the main events occurring during a trial and the corresponding color changes of the end target. In experiments 1 and 2, the feedback was dependent on the movement time, whereas in experiments 3 and 4, it was determined by the peak forward velocity. ***C***, Illustration of the random sequence of force-fields (counterclockwise: CCW, clockwise: CW) and visual gains (cursor gains) with null-field trials (NULL) interleaved. During null-field trials cursor and hand were always aligned (visual gain = 1). During perturbation trials, the *x*-coordinate of the cursor position was multiplied by a gain. ***D***, Overview of the four different experiments including the sample size (*N*), the task instruction (exp. 3: Reaching vs Shooting, exp. 4: Cursor vs. Hand) the magnitude of the force-field (small FF vs. large FF), and the visual gain levels. For clarity only the CCW direction of forces and gains are displayed here.

All four experiments were conducted using a KINARM Endpoint robotic device (KINARM). The experiments were designed using the MATLAB toolboxes Simulink and Stateflow (MATLAB 2015, MathWorks Inc.). Participants were instructed to grasp the right handle of the robotic device with their right hand and to perform visually guided, forward reaching movements of 15 or 12 cm in amplitude dependent on the experiment and the task instructions as specified below (Fig. 1*D*). The start and end targets of the reach (radii: 0.6 and 1.2 cm), as well as a hand-aligned cursor (radius: 0.5 cm) were projected onto the movement plane using a monitor mirror setup. Participants were seated so that the midline of their body was aligned with the midline of the monitor and their forearm was hidden from view during the entire experiment. To initiate a trial, participants had to move the hand-cursor into the start target, which was located 5 cm to the right from the midline and 10 cm upwards from the lower edge of the monitor. After a random interval of 2–4 s (uniformly distributed) the end target changed color, signaling the go-cue to start the movement (Fig. 1*B*).

Dependent on the experiment, we applied velocity-dependent force-fields orthogonal to the movement direction during 44–50% of all trials (Fig. 1*A*). During these force-field trials the lateral force applied to the hand was proportional to the forward velocity of the movement ( $f_{x}=\pm L\dot{y},L=13Nsm^{-1}$ or $L=25Nsm^{-1}$; see Fig. 1*D*). The direction of this force-field [clockwise (CW), or counterclockwise (CCW)] was randomized across trials. During these force-field trials, we applied an additional visual perturbation by multiplying the lateral position of the hand-cursor by a gain lesser or >1 to either decrease or increase the visually experienced lateral movement deviation (visual gain; Fig. 1*C*). In null-field trials the visual gain was always 1 so that the visual feedback was congruent with the true hand position.

The kinematic parameters of the movements were constrained either by allowing a specified time window to reach the goal target (experiments 1 and 2), or by imposing a certain maximum velocity range for the movements in experiments 3 and 4. This adaptation of the protocol was done because the different task instructions in experiments 3 and 4 produced different movement durations. The exact constraints applied to these parameters in each experiment are detailed below. After each movement participants received feedback about their performance, which was represented by a color-change of the end target. If the preceding movement was too slow, the target changed to a filled, red circle. If it was too fast, it changed to an open, red circle, and if the movement was correct, the target turned green (Fig. 1*B*). Participants scored one point for each correct movement and their incremented score was shown in the top right corner of the visual display. In the beginning of each experiment, participants were instructed to try to score as many points as they could to motivate them to comply with the task constraints. Following the feedback about movement success, the end target and the hand-cursor were extinguished and only reappeared once the participants’ hand moved within a 5-cm radius surrounding the start position. This was done to avoid visible cursor jumps between trials that might have informed participants about the visual gain manipulation applied to the hand-cursor. The four experiments differed in the values of visual gains used, in the task instructions, as well as in the strengths of the applied forces (Fig. 1*D*). In all cases, the total number of trials of each experiment was divided into several blocks. Participants were given the opportunity to rest between blocks. In the beginning of each experiment participants performed ten practice movements without perturbation to become acquainted with the task and timing constraints.

### Experiment 1

The aim of experiment 1 was to investigate the influence of combined force-field and visual perturbations on movement corrections and adaptation. Participants performed reaching movements during CW, CCW ( $L=\pm 13Nsm^{-1}$) and null force-fields in randomized order. Force perturbations were applied during 50% of all movements. During the CW and CCW force-field trials, one of five possible values of the visual gain (0, 0.5, 1, 1.5, 2) was applied randomly to manipulate the size of the visual error. A visual gain of 0 resulted in a situation in which the cursor moved straight to the target regardless of the lateral deviation of the hand. We chose this condition instead of completely removing the visual feedback because it has previously been shown that vision significantly influences movement correction particularly in circumstances where it requires a different response than proprioception or when it clearly contradicts proprioceptive feedback (Franklin and Wolpert, 2008; Kasuga et al., 2022). At the end of each movement participants received feedback based on their movement time. The trial was categorized as “correct” if the movement time was between 600 and 800 ms and participants were awarded one point for each correct trial. They performed a total of 600 trials divided into 10 blocks, each composed of 30 null-field trials and 30 perturbation trials distributed as follows: two force directions times five visual gains times three repetitions. This resulted in a total of 30 repetitions per force-field and visual gain combination per participant.

### Experiment 2

Experiment 2 aimed to assess the contribution of limb afferent feedback to motor adaptation in more detail. Specifically, we were interested to see whether single trial adaptation scales with the size of the force-field and whether the effect of the force-magnitude is modulated by the size of the visual error observed. Therefore, we used two different force-field strengths in combination with the visual gain manipulation (visual gains 0, 1, 2). In this experiment we chose only three values of the visual gain to reduce the total number of conditions. The smaller force-field was identical to that used in the previous experiment ( $L=\pm 13Nsm^{-1}$) and the larger force-field ( $L=\pm 25Nsm^{-1}$) was chosen such that there would be a clear difference in experienced hand deviations. As in experiment 1, trials were considered correct if the movement time was between 600 and 800 ms. Each participant performed a total of 656 trials divided into 8 blocks with force-field strength and directions randomized across trials. We pseudo-randomized the order of trials in such a way that each perturbation trial was followed by one or sometimes two null-field trials. This was done to increase the number of trials we could use to look at single-trial adaptation. Force perturbations were applied during 44% of all trials. Each block was composed of 46 null-field trials and 36 perturbation trials (two force directions times three visual gains times three repetitions times two force strengths). This resulted in 24 repetitions of each force-field and visual gain combination per participant.

### Experiment 3

Building up on experiments 1 and 2, the goal of experiment 3 was to investigate whether the instruction to correct for combined force and visual perturbations had an influence on how these two modalities contributed to online control and adaptation. To this end, we used similar force-field and visual gain perturbations as in experiment 1 and combined them with two different task instructions. Similar to experiment 2, we used only three values of the visual gain (0, 1, 2) and the same pseudo-randomized perturbation schedule. For the “reaching task”, we instructed participants, as in experiments 1 and 2, to correct their movements when they experienced a perturbation and try to stabilize the cursor inside the end target. Conversely, in the “shooting task”, we told participants to aim for the target and shoot through it, but to not correct their movements when they encountered a perturbation. In this case, they were instructed to let their hand be pushed away by the force-field. When the hand crossed a 12-cm distance from the start target the visual cursor was removed to prevent endpoint corrections. Participants performed the shooting and reaching tasks in a blocked fashion. The order in which the two instructions were performed was counterbalanced across them, resulting in two subgroups of 12 participants starting with one or the other instruction. To encourage consistent movement velocities across tasks, participants received feedback about the peak velocity of their movement instead of the movement time. Trials were considered “correct” if the peak forward velocity was between 0.5 and 0.8 m/s. During the shooting task, participants were awarded one point if their movement was within the specified velocity range independent on whether or not they hit the target. This was done to encourage them to comply with the instruction to not correct their movement during perturbation trials. To incentivize participants to try to shoot through the target when no force was applied, we awarded them two points instead of one if the cursor moved through the target on null-field trials (and the peak velocity of the movement was in the required range). Force perturbations were applied during ∼45% of all trials. Participants performed a total of 330 trials per task instruction divided into five blocks, each composed of 36 null-field trials and 30 perturbation trials distributed as follows: two force directions times three visual gains times five repetitions. This resulted in 25 repetitions of each force-field and visual gain combination per task instruction and participant.

### Experiment 4

Experiment 4 aimed to test whether explicitly instructing participants to either control the cursor or their hidden hand could influence the contribution of visual feedback to online control and adaptation. To this end, we used the reaching task from experiment 3 and added a second task instruction during which participants were informed that the cursor did not always accurately reflect their hand position and that they should try to stabilize their hidden hand inside the end target instead of the cursor. For the purpose of this experiment, we called the original instruction “cursor task” and the adapted instruction “hand task”. In contrast to the previous experiments, we used a different set of values of the visual gain. A gain value of 0 acts like a visual error clamp which strongly reduces feedback corrections. As we observed in previous experiments that it could be a special case, we wanted to use a low value of the gain which still requires a feedback response. For this reason, we chose 0.3, 1, and, by symmetry, 1.7 as values of the visual gain. In all other points the protocol of experiment 4 was identical to the reaching task of experiment 3. Importantly, participants also performed the two task instructions in a blocked fashion and the task order was counterbalanced across them.

### Kinematics: data collection and analysis

Kinematic data were collected using a sampling frequency of 1 kHz. The data preprocessing was performed using custom-written MATLAB code (MATLAB 2021a, MathWorks Inc.). All recordings were aligned to movement onset defined as the moment when the tangential velocity exceeded a threshold of 10% of its maximum value. Movement offset was defined as the first sample below a threshold of 0.01 m/s lateral hand velocity with the exception of the “shooting task” in experiment 3 were the movement offset was defined as the first sample exceeding a distance of 15 cm from the start target. All kinematic data were filtered using a low-pass fourth order, dual-pass Butterworth filter with a cut off frequency of 50 Hz. Hand velocity was computed offline using a fourth-order central difference approximation applied to the position data.

To determine the hand errors experienced during the perturbation trials, we computed the maximum hand deviation perpendicular to the movement direction (maximum error) as well as the lateral hand deviation averaged across a 100-ms window centered on the time of movement offset (endpoint error). Before computing the maximum and endpoint errors, the mean lateral deviation across all null-field trials was subtracted from the lateral position traces of all trials for each participant separately. This means that an error of zero signifies a lateral deviation comparable to baseline behavior. Hence, the maximum error approximately coincides with the time point when the visual perturbation was maximal, whereas the endpoint error captures the final correction of the hand close to the target. We derived the visually experienced cursor deviations offline by multiplying hand deviations by the visual gain. Lastly, to quantify the adaptation that occurred in response to the exposure to a single combination of force-field and visual gain, we looked at the maximum deviation of the hand during each null-field trial following directly after a force-field trial and subtracted the average deviation across all null-field trials for each participant separately (after-effect). Such a single trial after-effect indicates the impact of the experienced perturbation on the planning of the very next movement. Thus, it provides an estimate of the adaptation of the motor plan (Marko et al., 2012; Sing et al., 2013; Albert and Shadmehr, 2016). In addition, we performed a follow-up analysis to assess whether our original measure of the after-effect was significantly influenced by visual feedback corrections induced by the cursor-deviation during null-field trials. For this analysis we quantified after-effects as the first lateral force peak relative to the average lateral force across all null-field trials. We extracted these lateral force peaks between 50 and 150 ms after movement onset when the force clearly opposed the previously encountered force-field. Importantly, during perturbation trials, we observed changes in EMG activity corresponding to the visual gain only after 150 ms. Therefore, it is unlikely that these early force-peaks were dominated by visual feedback corrections.

### Muscle recordings: data collection and analysis

In experiments 1, 3, and 4 (data of experiment 4 not shown), we recorded muscle signals from the pectoralis major (PM) and posterior deltoid (PD) of the right shoulder which act as antagonists to the CW and CCW force-fields, respectively (Fig. 1*A*). Muscle activity (Electromyography, EMG) was collected using bipolar surface electrodes (DE-2.1 EMG Sensor, Delsys) attached to the muscle belly. Before attaching each electrode, the skin underneath was cleaned using cotton gauze and medical alcohol and the contacts of the electrodes were coated with conductive gel. The EMG signals were amplified by a factor of 1000 or 10,000, depending on the subject (Bagnoli-8 EMG System, Delsys) and recorded at a sampling frequency of 1 kHz.

All muscle recordings were aligned to movement onset and bandpass filtered using an eighth order, dual-pass Butterworth filter with cutoff frequencies below 20 Hz and above 250 Hz. In addition, we applied a notch filter at 50 Hz to remove environmental noise. After filtering, muscle recordings were rectified and normalized by the average activity measured against a constant force applied to the hand. This activity was measured in four separate calibration blocks which were performed after every two blocks of the main experiment. During these calibration blocks participants had to remain inside a square (2 × 2 cm) presented on the screen while countering a 5N force that was applied for 2 s to the right or left against their hand, corresponding to the lines of actions of the chosen muscles. Each of the two force directions was repeated three times per block in a randomized order. From each of these trials we extracted a 1 s recording between 0.5 and 1.5 s after the force was turned on and computed the average rectified muscle activity across time and repetitions of the same force-direction. Then, we divided all recordings of the PM muscle by the calibration recordings measured against a rightward force in the same muscle, and all PD recordings by the calibration trials against a leftward force.

Since we were interested in the change in muscle activity because of the combined force and visual perturbations, we subtracted the average null-field activity of each muscle from all of the recordings made during the perturbation trials. To illustrate the effect of the visual gain on muscle activities we computed the group-average time series of each value of the visual gain in the agonist and antagonist muscles separately for both force-field directions. Next, to perform more detailed statistical analyses, we computed the average muscle responses across a 200-ms time window for each participant in every visual gain condition. For the agonist muscle this time window was set to 150–350 ms following movement start (early EMG) and for the antagonist muscle to 350–550 ms following movement start (late EMG). We chose these time windows based on visual inspection of the average EMG traces such that they corresponded to the time when the muscle activity clearly separated depending on the visual gain condition.

### Statistical design

All statistical analyses were performed using custom written MATLAB scripts (MATLAB 2021a, MathWorks Inc.). We conducted all statistical tests separately for CCW and CW force-fields. For experiment 1, we used linear mixed effect models (LMEs) with participant ID as a random factor to assess the global effect of the visual gain (VG) on movement errors, EMG responses, and after-effects. In our models, we defined the visual gain as a continuous predictor variable:

$$y_{i,j}=\beta_{0}+\beta_{1}VG+b_{j}+\epsilon_{i,j}.$$

Here, $y_{i,j}$ represents the ith observation in the dependent variable (movement error, EMG response, or after-effect) for participant j, $\beta_{0}$ represents the group-level intercept, $\beta_{1}$ is the slope-estimate for the visual gain, $b_{j}$ is the random intercept for participant j, and $\epsilon_{i,j}$ represents the residual for observation i of participant j. For experiments 2, 3, and 4, we fitted an LME that included the force-field strength (experiment 2) or the task instruction (experiments 3 and 4) and the interaction with the visual gain as additional fixed effects. In those cases, force-field strength or task instruction were defined as a factor with two levels and the first level was used as reference category. For each significant LME result we reported the estimated slope or intercept as well as the 95% confidence interval of the estimate (CI) in the statistical table (Table 1). All LME models were fitted using maximum likelihood estimation. Before fitting the LME models to our data, we tested whether the data were normally distributed on the individual participant level and within every level of the predictor variables using Lilliefors test. The first column of the statistical table reports the percentage of cases in which the data followed a normal distribution.

For experiment 3, we performed a follow-up analysis in which we compared the slope estimates for maximum hand errors and visual gain between the reaching and shooting task. To this end, we estimated the distributions of slope parameters using bootstrapping and compared the means of the distributions using paired *t* tests. For the bootstrapping analysis we sampled 1000 times with repetition from our sample of participants and computed at each iteration the effect of the maximum hand error and visual gain using an LME with both predictors as fixed effects, but excluding the interaction term.

We computed *post hoc* pairwise comparisons with Bonferroni corrections to illustrate interesting differences in behavior between visual gain conditions, force-field strengths or tasks. For all relevant comparisons we reported Cohen’s *d*_av_ as a measure of effect size (Lakens, 2013):

$$Cohen^{\prime }s\;d_{av}=\frac{M_{Diff}}{\frac{SD_{1}\;+\;SD_{2}}{2}}.$$

M_Diff_ represents the mean difference between conditions and SD_i_ the standard deviation of condition i.

Finally, to assess the relationship between maximum hand errors and after-effects on the subsequent trial, we computed regression slopes for each participant separately for each task/force-field strength and each visual gain condition. These regressions were computed based on the z-transformed values of the maximum hand errors and after-effects. Z-scores were computed for each participant relative to the mean in the visual gain 1 condition. Throughout this paper, all results with a (Bonferroni-corrected) *p*-value below 0.05 are considered significant.

## Results

### Experiment 1: scaling of visual error influences online corrections but not adaptation

In the first experiment, we investigated whether online corrections and adaptation to random force-field perturbations scaled with the size of the visual cursor displacement. To this end, participants (*N* = 14) were exposed to a sequence of CW and CCW velocity-dependent force-fields randomly interleaved with null-field trials. During force-field trials we increased or decreased the lateral deviation of the cursor by applying a gain (visual gain) to manipulate the visual errors observed during the perturbation.

**Figure 2.**
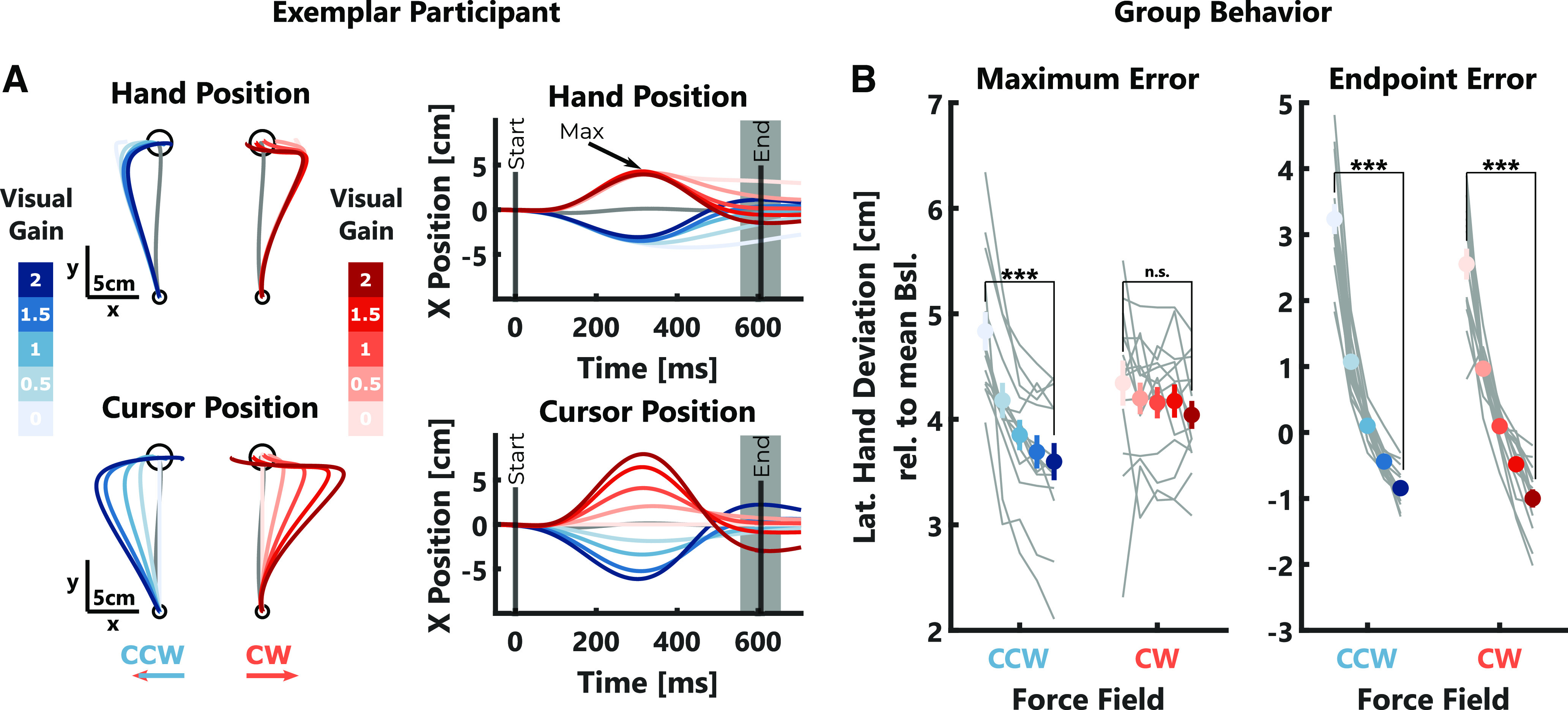
Behavioral data experiment 1. ***A***, Left, Mean hand trajectories (top) and cursor trajectories (bottom) in *x*- and *y*-dimensions for one exemplar participant. Shades of blue and red indicate the different visual gain conditions for counterclockwise (CCW) and clockwise (CW) trials, respectively. The gray trace shows the average trajectory during null-field trials. Right, Mean hand (top) and cursor (bottom) *x*-positions over time for the same participant. The black arrow indicates when maximum hand errors were computed (Max). The median movement duration during force-field trials is indicated by a black vertical line (End). The gray-shaded area shows the 100-ms time window over which the endpoint error was computed. ***B***, Group averages (±SEM) of maximum (left) and endpoint (right) hand errors are shown in color and individual participant means are shown as gray lines. Note, that values for CCW perturbations were inverted to positive values for better illustration. Stars indicate significant differences determined using paired comparisons with *post hoc* Bonferroni corrections: ^n.s^*p* > 0.05, ***p < 0.0001.

We observed that participants corrected their movements more strongly with increasing visual gain, which was mostly visible toward the end of the reach (Fig. 2*A*). To compare the behavior across participants, we extracted the maximum deviation of the hand lateral to the movement direction (maximum error) as well as the lateral deviation of the hand averaged across a 100 ms window centered around the end of the movement (endpoint error). Our results show that both of these errors decreased significantly with increasing visual gain (Fig. 2*B*; maximum error: CW: *t*_(2067)_ = −3.38, *p* = 0.0007; CCW: *t*_(2062)_ = −20.84, *p* < 10^−4^; see Table 1, a; endpoint error: CW: *t*_(2067)_ = −49.64, *p* < 10^−4^; CCW: *t*_(2062)_ = −61.03, *p* < 10^−4^; see Table 1, b). We verified that the visual gain did not systematically influence the experienced force perturbation. As there was no relationship between the visual gain and the maximum forward velocity, we can conclude that the observed decrease in hand errors with the visual gain is because of an increase in feedback corrections.

**Table 1:** Statistical table

	**Data structure**	**Type of test**	**Power**	
**Experiment 1**				
	Data tested for normality using Lilliefors test on individual participant level (α = 0.01)	Linear-mixed model Fixed effect: visual gain		
		Random effect: participant ID		
		**Dependent variable**	**CCW**	**CW**
a	95% not significantly different from normal distribution	Maximum error	Slope = −0.59 CI = [−0.65, −0.54]	Slope = −0.12 CI = [−0.2, −0.05]
b	90% not significantly different from normal distribution	Endpoint error	Slope = −1.94 CI = [−2, −1.87]	Slope = −1.71 CI = [−1.78, −1.64]
c	91% not significantly different from normal distribution	Early EMG	Slope = 0.23 CI = [0.2, 0.26]	Slope = 0.12 CI = [0.1, 0.14]
d	79% not significantly different from normal distribution	Late EMG	Slope = 0.26 CI = [0.24, 0.27]	Slope = 0.3 CI = [0.26, 0.33]
e	97% not significantly different from normal distribution	After-effect	Not significant	Not significant
	92% not significantly different from normal distribution	After-effect (lat. force)	Not significant	Not significant
**Experiment 2**				
	Data tested for normality using Lilliefors test on individual participan level (α = 0.01)	Linear-mixed model Fixed effects: visual gain, force-field strength,interaction		
		Random effect: participant ID		
		**Dependent variable**	**CCW**	**CW**
f	Small FF: 99%; large FF: 92% not significantly different from normaldistribution	Maximum error	Main effect visual gain	
			Slope = −0.94 CI = [−1.05,−0.83]	Slope = −0.42 CI = [−0.56,−0.28]
			Main effect FF strength	
			Intercept = 4.12 CI = [3.92,4.32]	Intercept = 5.48 CI = [5.23,5.74]
			Interaction	
			Slope = −0.46 CI = [−0.61,−0.31]	Slope = −0.64 CI = [−0.84,−0.45]
g	Small FF: 95%; large FF: 90% not significantly different from normaldistribution	Endpoint error	Main effect visual gain	
			Slope = −2.43 CI = [−2.57,−2.3]	Slope = −2.31 CI = [−2.48,−2.14]
			Main effect FF strength	
			Intercept = 3.33 CI = [3.08,3.58]	Intercept = 4.21 CI = [3.9,4.53]
			Interaction	
			Slope = −1.77 CI = [−1.97,−1.58]	Slope = −2.16 CI = [−2.41,−1.92]
h	Small FF: 95%; large FF: 94% not significantly different from normal distribution	After-effect	Main effect visual gain	
			Slope = 0.08 CI = [0.04,0.12]	Not significant
			Interaction	
			Slope = 0.06 CI = [0.01,0.11]	Not significant
	Small FF: 95%; large FF: 97% not significantly different from normal distribution	After-effect (lat. force)	Main effect visual gain	
			Slope = 0.06 CI = [0.03,0.09]	Slope = 0.04 CI = [0.01,0.06]
			Main effect FF strength	
			Intercept = 0.09 CI = [0.04,0.15]	Not significant
**Experiment 3**				

	Data tested for normality using Lilliefors test on individual participant level (α = 0.01)	Linear-mixed model Fixed effects: visual gain, task, interaction		
		Random effect: participant ID		
		**Dependent variable**	**CCW**	**CW**
j	Reaching: 97%; shooting: 97% not significantly different from normal distribution	Maximum error	Main effect visual gain	
			Slope = −0.61 CI = [−0.67,−0.56]	Slope = −0.25 CI = [−0.33,−0.17]
			Interaction	
			Slope = 0.51 CI = [0.43,0.59]	Slope = 0.29 CI = [0.18,0.41]
k	Reaching: 94%; shooting: 97% not significantly different from normal distribution	Endpoint error	Main effect visual gain	
			Slope = −2.68 CI = [−2.74,−2.61]	Slope = −2.7 CI = [−2.79,−2.62]
			Interaction	
			Slope = 2.57 CI = [2.47,2.66]	Slope = 2.74 CI = [2.62,2.86]
l	Reaching: 92%; shooting: 74% not significantly different from normal distribution	Early EMG	Main effect visual gain	
			Slope = 0.28 CI = [0.24,0.32]	Slope = 0.2 CI = [0.17,0.22]
			Main effect task	
			Intercept = −0.34 CI = [−0.41,−0.27]	Intercept = −0.27 CI = [−0.32,−0.23]
			Interaction	
			Slope = −0.25 CI = [−0.3,−0.19]	Slope = −0.13 CI = [−0.17,−0.1]
m	Reaching: 85%; shooting: 60% not significantly different from normal distribution	Late EMG	Main effect visual gain	
			Slope = 0.28 CI = [0.26,0.3]	Slope = 0.42 CI = [0.38,0.45]
			Main effect task	
			Intercept = −0.1 CI = [−0.11,−0.04]	Intercept = −0.18 CI = [−0.24,−0.12]
			Interaction	
			Slope = −0.27 CI = [−0.3,−0.24]	Slope = −0.4 CI = [−0.45,−0.36]
n	Reaching: 94%; shooting: 92% not significantly different from normal distribution	After-effect	Main effect visual gain	
			Slope = 0.11 CI = [0.07,0.14]	Slope = 0.07 CI = [0.04,0.1]
	Reaching: 95%; shooting: 97% not significantly different from normal distribution		After-effect (lat. force)	Main effect visual gain
			Slope = 0.05 CI = [0.02,0.07]	Slope = 0.04 CI = [0.01,0.07]
**Experiment 4**				
	Data tested for normality using Lilliefors test on individual participant level (α = 0.01)	Linear-mixed model Fixed effects: visual gain, task, interaction		
		Random effect: participant ID		
		**Dependent variable**	**CCW**	**CW**
o	Cursor: 100%; hand: 99% not significantly different from normal distribution	Maximum error	Main effect visual gain	
			Slope = −0.34 CI = [−0.42, −0.27]	Slope = −0.12 CI = [−0.22, −0.02]
p	Cursor: 91%; hand: 92% not significantly different from normal distribution	Endpoint error	Main effect visual gain	
			Slope = −1.37 CI = [−1.44, −1.3]	Slope = −1.49 CI = [−1.57, −1.41]
			Main effect task	
			Intercept = −0.4 CI = [−0.51, −0.28]	Intercept = −0.41 CI = [−0.54, −0.27]
			Interaction	
			Slope = 0.15 CI = [0.06, 0.25]	Slope = 0.2 CI = [0.09, 0.32]
q	Cursor: 99%; hand: 95% not significantly different from normal distribution	After-effect	Main effect visual gain	
			Slope = 0.07 CI = [0.02, 0.12]	Slope = 0.05 CI = [0.005, 0.09]
			Main effect task	
			Intercept = 0.09 CI = [0.01, 0.17]	Not significant
	Cursor: 99%; hand: 97% not significantly different from normal distribution	After-effect (lat. force)	Main effect visual gain	
			Not significant	Slope = 0.05 CI = [0.01, 0.09]
			Main effect task	
			Intercept = 0.07 CI = [0.01, 0.14]	Not significant

CCW = counterclockwise perturbation, CW = clockwise perturbation, CI = 95% confidence interval of the slope/intercept estimate, EMG = electromyography.

**Figure 3.**
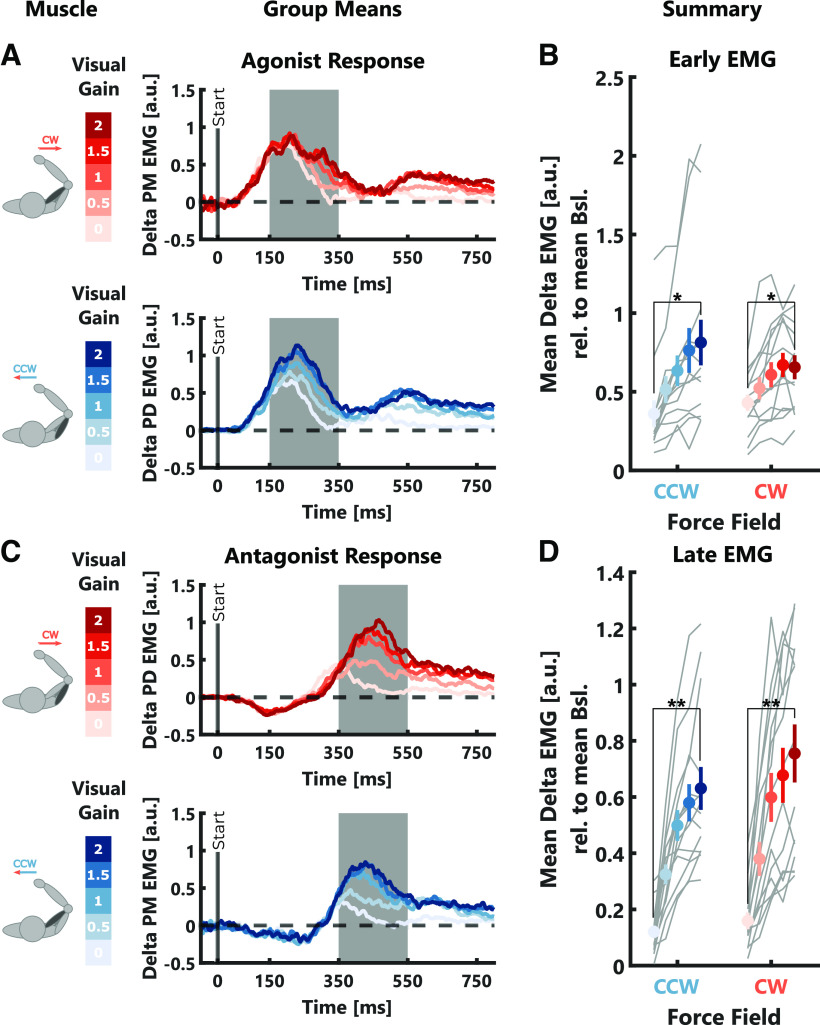
EMG data experiment 1. ***A***, Group average δ EMGs (relative to average activity during null-field trials) of the agonist muscle for the two force-field directions. For illustrative reasons, the depicted traces were smoothed using a centered moving average with a window size of 10 samples. The gray-shaded areas indicate the time window across which early muscle responses were computed. ***B***, Group average early muscle responses ± SEM are shown in color. Individual participant means are shown as gray lines. ***C***, Same as ***A*** but for the antagonist muscle. In this case, the gray-shaded areas indicate the time window across which the late muscle responses were computed. ***D***, Same as ***B*** but for late muscle responses measured in the antagonist muscle. The color-code is the same as in Figure 2. Stars indicate significant differences determined using paired comparisons with *post hoc* Bonferroni corrections: *p < 0.05, **p < 0.001.

This modulation of corrective responses with the size of the visual gain was also reflected in the muscle activities we measured in the pectoralis major (PM) and the posterior deltoid (PD) muscles of the shoulder. The early agonist responses, averaged across a time window of 150–350 ms following movement onset, significantly increased with increasing visual gain (Fig. 3*A*,*B*; CW: *t*_(2067)_ = 9.93, *p* < 10^−4^; CCW: *t*_(2062)_ = 14.90, *p* < 10^−4^; see Table 1, c). Similarly, the later antagonist responses, measured between 350 and 550 ms after movement onset, also scaled with the size of the visual gain (Fig. 3*C*,*D*; CW: *t*_(2067)_ = 17.12, *p* < 10^−4^; CCW: *t*_(2062)_ = 25.69, *p* < 10^−4^; see Table 1, d). These results, together with the kinematic results presented above, show that visual information was clearly used to perform online corrections during the movement.

**Figure 4.**
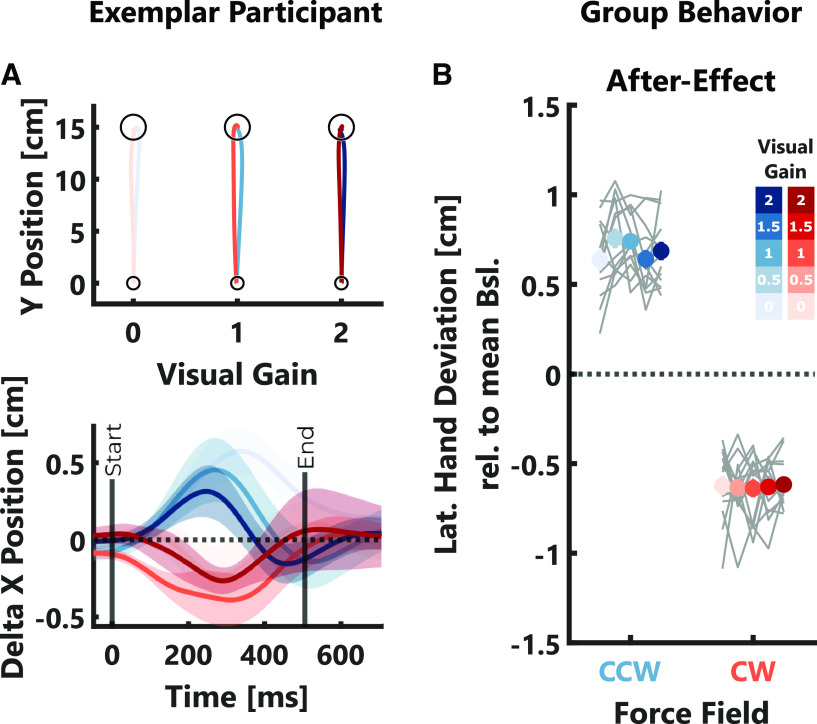
Adaptation data experiment 1. ***A***, Top, Mean hand trajectories during null-field trials immediately following three selected force-field direction and visual gain combinations for one exemplar participant. Bottom, Mean x-positions over time during the same null-field trials and for the same exemplar participant as above. Shading indicates ± SEM and median movement duration during null-field trials is indicated by a black vertical line (End). Please note, the *x*-positions shown were computed relative to the average across all null-field trials. ***B***, Group averages of after-effects are shown in color and gray lines indicate individual participant means. The color code is identical to Figure 2.

The observation that corrective responses during perturbation scale with the visual gain raised the question whether the size of the visual error also influenced the amount of adaptation to the force-field on the subsequent movement. To address this question, we quantified after-effects as the maximum lateral deviation relative to the average baseline behavior during null-field trials following immediately after a perturbation trial (Fig. 4). We observed clear after-effects in the opposite direction of the preceding force-field (one-tailed *t* test; CW: *t*_(13)_ = −23.89, *p* < 10^−4^; CCW: *t*_(13)_ = 17.44, *p* < 10^−4^). However, these after-effects were not influenced by the size of the visual error experienced during the preceding trial (CW: *t*_(1036)_ = 0.01, *p* > 0.05; CCW: *t*_(1031)_ = −0.21, *p* > 0.05; see Table 1, e). Given that the maximum lateral deviation during the null-field trials occurred around 300 ms after movement onset (Fig. 4*A*), it is possible that this measure is influenced by feedback corrections to the visual cursor displayed during the null-field trial itself. If so, this might have obscured the influence of the previously encountered visual error on adaptation. To assess whether our analysis accurately captured any potential influence of the visual gain on adaptation, we extracted the peak lateral force relative to the average baseline between 50 and 150 ms following movement onset when the force clearly opposed the previously experienced force-field (data not shown here). Importantly, during the perturbation trials, we observed visual gain-related changes in EMG activity only after 150 ms (Fig. 3*A*), therefore these early force peaks are unlikely influenced by visual feedback corrections. In this follow-up analysis, we also observed no effect of the visual gain on the early lateral forces (CW: *t*_(1036)_ = −0.7, *p* > 0.05; CCW: *t*_(1031)_ = 1.36, *p* > 0.05; see Table 1, e). Thus, we conclude that while visual feedback influenced online corrections, it did not carry over to adapt the next movement.

Taken together, the results of our first experiment show that participants used visual feedback to correct movements during force-field perturbation. However, subsequent adaptive responses did not increase with the size of the visual error. Importantly, the after-effects observed with a random force-field paradigm are very small and the exposure to several different perturbation trials in a row might have interfered with the effect of the visual gain. To consider these possibilities we conducted a second experiment during which each perturbation trial was followed by a null-field trial to assess after-effects. Additionally, it was necessary to verify that the absence of scaling of after-effects with visual error size was not because of a ceiling or saturation effect in adaptation linked to the random perturbation schedule. Thus, we used two different strengths of force-fields to see whether adaptation increases with perturbation strength.

### Experiment 2: eliminating perpendicular visual errors reduces adaptation independent of force-field strength

Our second experiment aimed to test whether single trial adaptation scaled with different force-field magnitudes, and whether this scaling was further modulated by the size of the visual error. Sixteen participants performed reaching movements with randomly applied combinations of force-field directions and visual gains. Contrary to experiment 1, each force-field trial was followed by a null-field trial which was used to extract after-effects. This pseudo-randomized perturbation schedule was used to increase the number of trials during which we could observe an after-effect and to reduce interference between different perturbation trials on the after-effect. In addition, the strength of the force-field (FF) applied (small vs large) varied randomly between trials.

**Figure 5.**
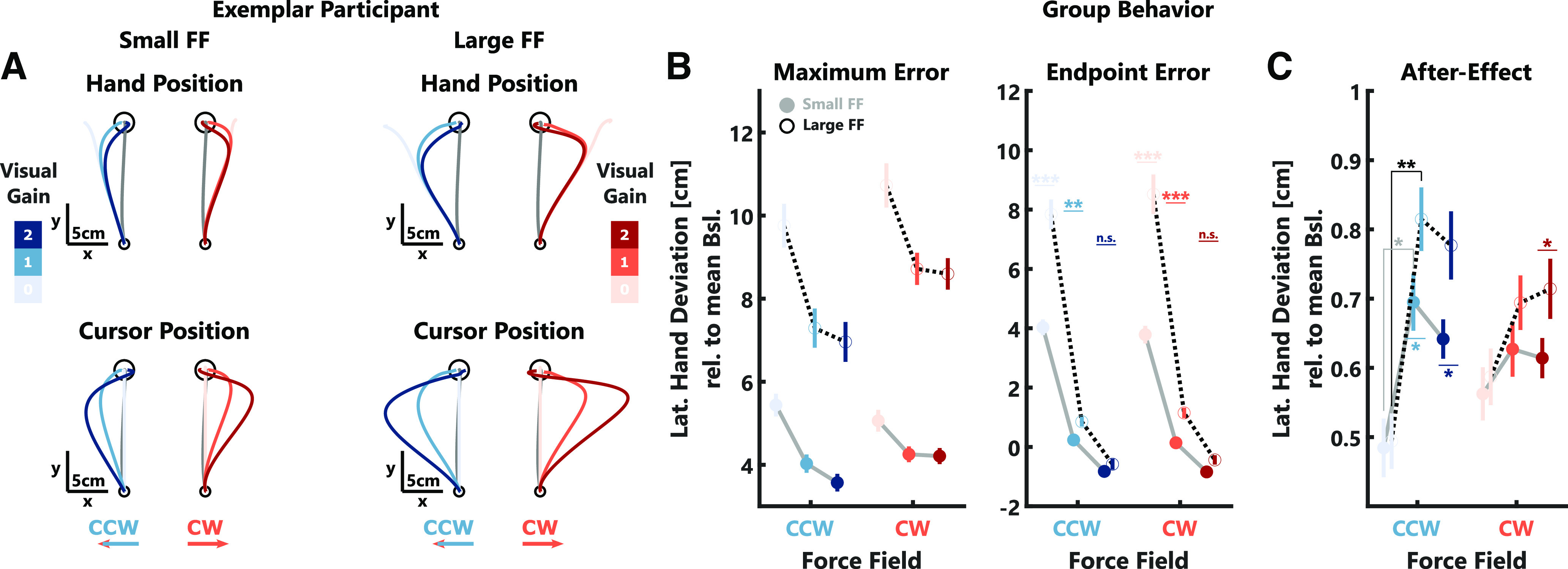
Behavioral and adaptation data experiment 2. ***A***, Mean hand (top) and cursor (bottom) trajectories for one exemplar participant during small (left) and large force-field trials (right). ***B***, Group average maximum (left) and endpoint errors (right) ± SEM. Note, that values for CCW perturbations were inverted to positive values for better illustration. The small force-field trials are represented by gray lines and filled circles, the large force-field trials by black-dotted lines and open circles. ***C***, Group average after-effects. Note, that values for CW perturbations were inverted to positive values for better illustration. The color-code follows the explanation in Figure 2. Stars indicate significant differences determined using paired comparisons with *post hoc* Bonferroni corrections: ^n.s^*p* > 0.05, *p < 0.05, **p < 0.001, ***p < 0.0001.

Similar to experiment 1, we observed a decrease of maximum and endpoint hand errors with increasing visual gain in both force-field strength conditions (Fig. 5*A*,*B*; maximum error: CW: *t*_(2276)_ = −5.9, *p* < 10^−4^; CCW: *t*_(2274)_ = −16.9, *p* < 10^−4^; see Table 1, f; endpoint error: CW: *t*_(2276)_ = −26.18, *p* < 10^−4^; CCW: *t*_(2274)_ = −34.71, *p* < 10^−4^; see Table 1, g). Further, we found that maximum and endpoint hand errors were larger during larger force-fields (maximum error*:* CW: *t*_(2276)_ = 41.98, *p* < 10^−4^; CCW: *t*_(2274)_ = 40.69, *p* < 10^−4^; see Table 1, f; endpoint error*:* CW: *t*_(2276)_ = 26.13, *p* < 10^−4^; CCW: *t*_(2274)_ = 26.05, *p* < 10^−4^; see Table 1, g) and that they decreased more strongly with the size of the visual error (maximum error: CW: *t*_(2276)_ = −6.36, *p* < 10^−4^; CCW: *t*_(2274)_ = −5.87, *p* < 10^−4^; see Table 1, f; endpoint error*:* CW: *t*_(2276)_ = −17.32, *p* < 10^−4^, CCW: *t*_(2274)_ = −17.86, *p* < 10^−4^; see Table 1, g).

Next, we investigated whether after-effects were modulated by the strength of the force-field and the visual gain (Fig. 5*C*). Contrary to experiment 1, some small differences in after-effects were visible depending on the visual gain. During CCW trials, we observed an increase in after-effects with the visual gain (*t*_(2275)_ = 4.23, *p* < 10^−4^; see Table 1, h) as well as a small interaction between the visual gain and the strength of the force-field (*t*_(2275)_ = 2.3, *p* = 0.0218; see Table 1, h). Importantly, we observed a significant decrease in after-effects between visual gain 1 and 0 during CCW trials for both force-field strengths (small FF: *t*_(15)_ = −4.33, *p* = 0.0018, Cohen’s *d*_av_ = −1.26; large FF: *t*_(15)_ = −4.96, *p* = 0.0005, Cohen’s *d*_av_ = −1.86), but no difference in after-effects between visual gain 1 and 2. Additionally, while after-effects increased with force-field strength for visual gain 1 (*t*_(15)_ = 2.32, *p* = 0.0348, Cohen’s *d*_av_ = 0.69) and 2 (*t*_(15)_ = 3.52, *p* = 0.0031, Cohen’s *d*_av_ = 0.87), this difference was eliminated when the visual gain was 0. The trend was qualitatively similar for CW trials; however, the after-effects only differed significantly between small and large force-fields for trials with visual gain 2 (*t*_(15)_ = 2.51, *p* = 0.0241, Cohen’s *d*_av_ = 0.69). When we quantified the after-effect as the early lateral force peaks during null-field trials, we also obtained an increase in adaptation with the size of the visual gain (CW: *t*_(2274)_ = 2.92, *p* = 0.0035; CCW: *t*_(2275)_ = 3.97, *p* = 0.0001; see Table 1, h). Further, during CCW trials, the lateral forces during null-field trials were slightly larger following large force-fields (*t*_(2275)_ = 3.45, *p* = 0.0006; see Table 1, h).

The observation that after-effects increased with force-field strength suggests, that the absence of scaling of after-effects with increasing visual errors in experiment 1 was not because of a saturation or ceiling effect of adaptation. Interestingly, when visual deviations were removed, participants exhibited similar after-effects across both force-field strengths. Thus, visual feedback had a conditional influence on adaptation that differed from the size-dependent scaling observed during feedback corrections. The observation, that visual feedback reduced after-effects precisely when it eliminated the need to correct for perturbations of the hand, raises the question whether eliminating the need for feedback corrections altogether would further reduce or eliminate the contribution of visual feedback to adaptation.

### Experiment 3: explicit instruction to not correct for errors influences contribution of visual feedback to online control but not to adaptation

To build on the findings of the previous experiment, our third experiment aimed to investigate whether instructing participants to correct their movements during perturbation and to stabilize the cursor inside the target influences the way visual feedback contributes to online control and adaptation. We asked a group of 24 participants to perform reaching movements during similar combined force-field and visual gain perturbations using two different tasks. In the reaching task we instructed participants to correct for the perturbation and to stabilize the cursor inside the target, whereas in the shooting task, participants were told not to respond to the perturbation and instead to let their hand be pushed away by the force. We used a similar pseudo-randomized perturbation schedule as in experiment 2 to increase the number of movements during which we could observe an after-effect.

**Figure 6.**
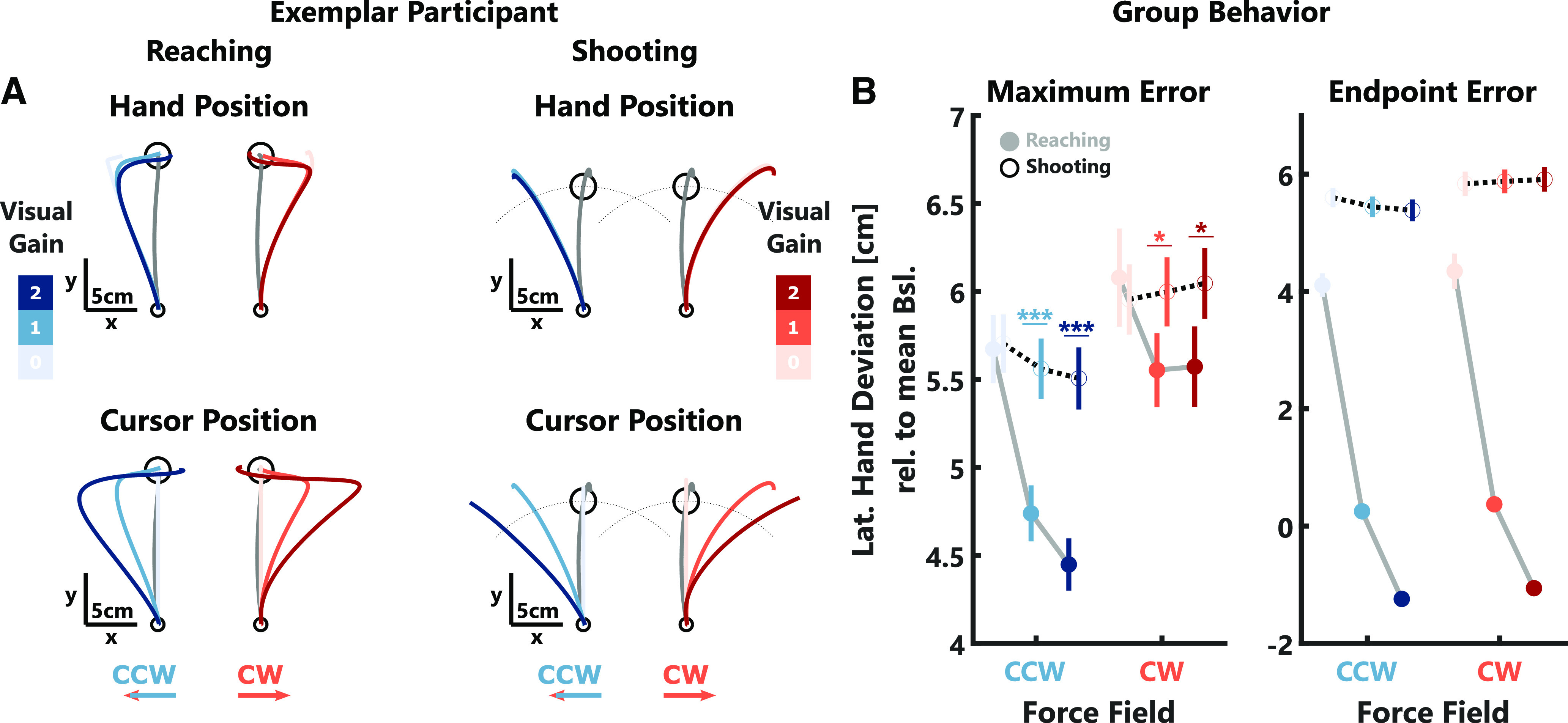
Behavioral data experiment 3. ***A***, Mean hand (top) and cursor (bottom) trajectories for one exemplar participant in the reaching (left) and shooting tasks (right). For the shooting task the black-dotted half-circles indicate a 12-cm radius from the start location. When this radius was crossed, the cursor disappeared to prevent feedback corrections. ***B***, Group average maximum (left) and endpoint errors (right) ± SEM. Note that values for CCW perturbations were inverted to positive values for better illustration. The reaching task is represented by gray lines and filled-circles, the shooting task by black-dotted lines and open circles. The color-code is the same as in Figure 2. Stars indicate significant differences determined using paired comparisons with *post hoc* Bonferroni corrections: *p < 0.05, ***p < 0.0001. See supporting Extended Data Figure 6-1 for an illustration of the data separated by the order in which the tasks were performed.

In line with experiments 1 and 2, we observed a decrease in maximum and endpoint hand errors with increasing visual gain (maximum error: CW: *t*_(3554)_ = −6.23, *p* < 10^−4^; CCW: *t*_(3550)_ = −21.43, *p* < 10^−4^; see Table 1, j; endpoint error: CW: *t*_(3554)_ = −60.99, *p* < 10^−4^; CCW: *t*_(3550)_ = −78.31, *p* < 10^−4^; see Table 1, k). While this decrease could clearly be observed in the reaching task, it was almost absent in the shooting task (Fig. 6). The results of the linear mixed model showed a significant interaction between visual gain and task instruction on both types of errors (maximum error: CW: *t*_(3554)_ = 5.18, *p* < 10^−4^; CCW: *t*_(3550)_ = 12.73, *p* < 10^−4^; see Table 1, j; endpoint error: CW: *t*_(3554)_ = 43.68, *p* < 10^−4^; CCW: *t*_(3550)_ = 53.16, *p* < 10^−4^; see Table 1, k). Using *post hoc* pairwise comparisons, we observed significantly smaller maximum hand errors in the reaching task compared with the shooting task in the visual gain 1 (CW: *t*_(23)_ = −2.76, *p* = 0.0111, Cohen’s *d*_av_ = −0.45; CCW: *t*_(23)_ = −6.24, *p* < 10^−4^, Cohen’s *d*_av_ = −1.01) and 2 conditions (CW: *t*_(23)_ = −2.88, *p* = 0.0084, Cohen’s *d*_av_ = −0.45; CCW: *t*_(23)_ = −7.85, *p* < 10^−4^, Cohen’s *d*_av_ = −1.33). Endpoint hand errors were significantly smaller in the reaching task for all visual gain conditions (all *p* < 10^−4^).

10.1523/ENEURO.0068-23.2023.f6-1 Extended Data Figure 6-1 Experiment 3: effect of task order. ***A***, Group average maximum hand errors for experiment 3 separated by task-order. The left panel shows the data of participants that started with the reaching task, the right panel shows the data for the group that started with the shooting task. Note that values for CCW perturbations were inverted to positive values for better illustration. The reaching task is represented by grey lines with filled-circles and the shooting task by black-dotted lines with open circles. ***B***, Same as ***A*** but for the endpoint hand error. Note, that values for CCW perturbations were inverted to positive values for better illustration. The color-code is identical to Figure 2. Download Figure 6-1, EPS file.

Given that all participants performed both task instructions in direct succession, it is possible that the order in which the tasks were performed had an influence on movement corrections. To investigate whether this was the case, we split the set of participants into two groups depending on the order in which they performed the tasks. We observed similar differences in maximum and endpoint errors between the reaching and shooting task for both groups of participants (Extended Data Fig. 6-1). Hence, online corrections were qualitatively similar independent of the order in which the tasks were performed.

**Figure 7.**
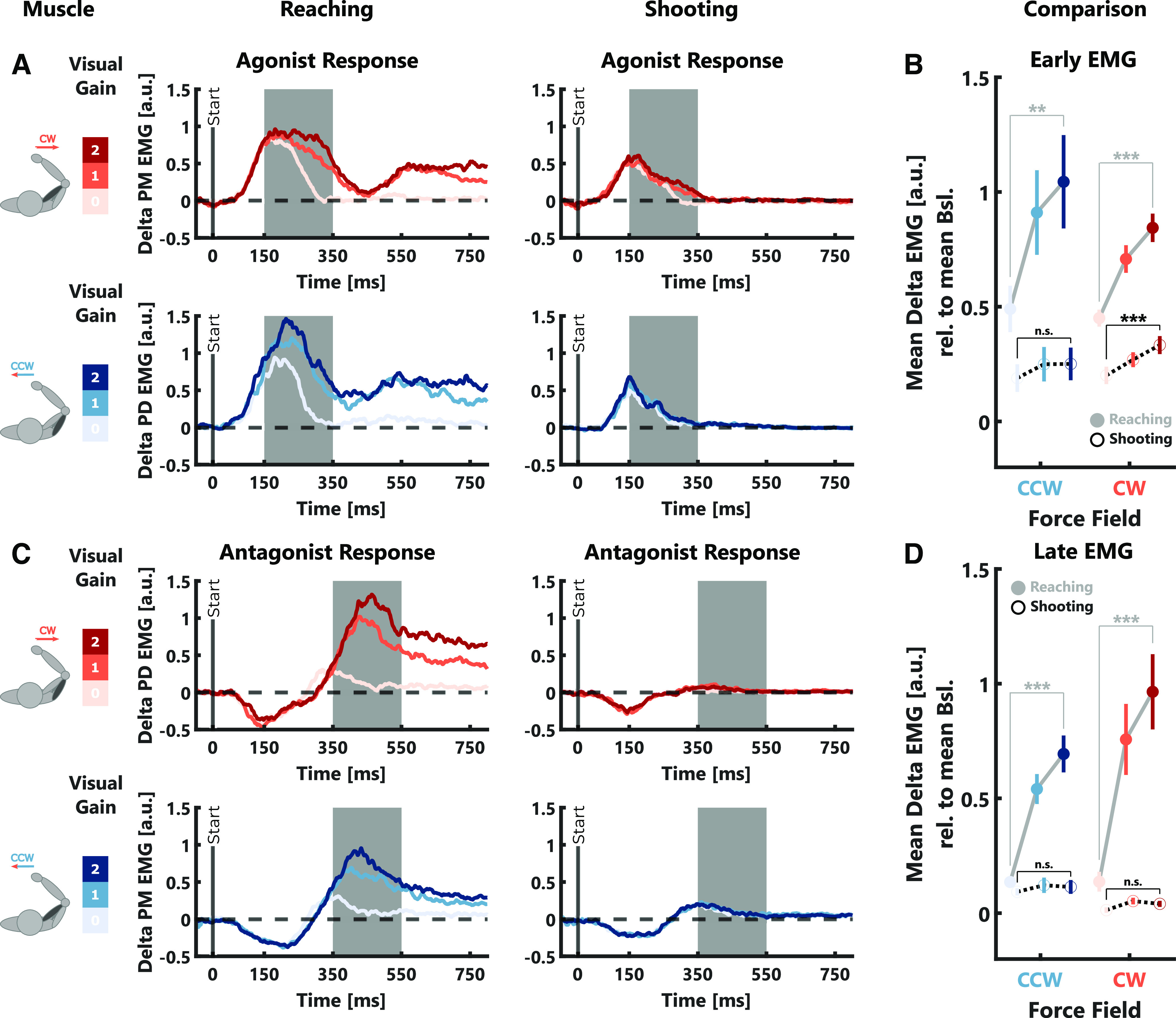
EMG Data experiment 3. ***A***, Group average δ EMGs (relative to average null-field activity) of the agonist muscle for the two force-field directions in the reaching (left) and shooting task (right). For illustrative reasons, the depicted traces were smoothed using a moving average with a window size of 10 samples. The gray-shaded areas indicate the time window across which early muscle responses were computed. ***B***, Group average early muscle responses ± SEM. The reaching task is represented by gray lines and filled circles, the shooting task by black-dotted lines and open circles. ***C***, Same as ***A*** but for the antagonist muscle. In this case, the gray-shaded areas indicate the time window across which the late muscle responses were computed. ***D***, Same as ***B*** but for late muscle responses measured in the antagonist muscle. The color-code is the same as in Figure 2. Stars indicate significant differences determined using paired comparisons with *post hoc* Bonferroni corrections: ^n.s^*p* > 0.05, **p < 0.001, ***p < 0.0001.

The different levels of visual contribution to online correction in the reaching and shooting tasks were also reflected in the early and late muscle responses (Fig. 7). Our statistical analysis showed a significant increase in muscle activity in early and late time intervals with increasing visual gain (early: CW: *t*_(3554)_ = 15.63, *p* < 10^−4^; CCW: *t*_(3550)_ = 13.97, *p* < 10^−4^; see Table 1, l; late: CW: *t*_(3554)_ = 25.15, *p* < 10^−4^; CCW: *t*_(3550)_ = 28.45, *p* < 10^−4^; see Table 1, m). This increase was stronger in the reaching compared with the shooting task (early: CW: *t*_(3554)_ = −7.34, *p* < 10^−4^; CCW: *t*_(3550)_ = −8.75, *p* < 10^−4^; see Table 1, l; late: CW: *t*_(3554)_ = −17.18, *p* < 10^−4^; CCW: *t*_(3550)_ = −19.44, *p* < 10^−4^; see Table 1, m) and overall muscle activities were significantly lower in the shooting compared with the reaching task (early: CW: *t*_(3554)_ = −11.73, *p* < 10^−4^; CCW: *t*_(3550)_ = −9.3, *p* < 10^−4^; see Table 1, l; late: CW: *t*_(3554)_ = −6.08, *p* < 10^−4^; CCW: *t*_(3550)_ = −4.33, *p* < 10^−4^; see Table 1, m). In the shooting task, *post hoc* comparisons revealed a significant increase in muscle activity between visual gain 0 and 2 only for early responses during CW trials (*t*_(23)_ = −3.34, *p* = 0.0086, Cohen’s *d*_av_ = −0.4), whereas in the reaching task there was a significant increase in both early and late intervals during both force-field directions (all *p* < 10^−4^). This residual response to the visual error during the early time interval in the shooting task might be the result of automatic perturbation responses which are known to include both limb and visual motor systems (Scott, 2016). Importantly, this effect was much smaller compared with the reaching task, demonstrating a very limited influence of visual feedback on movement corrections in the shooting task.

**Figure 8.**
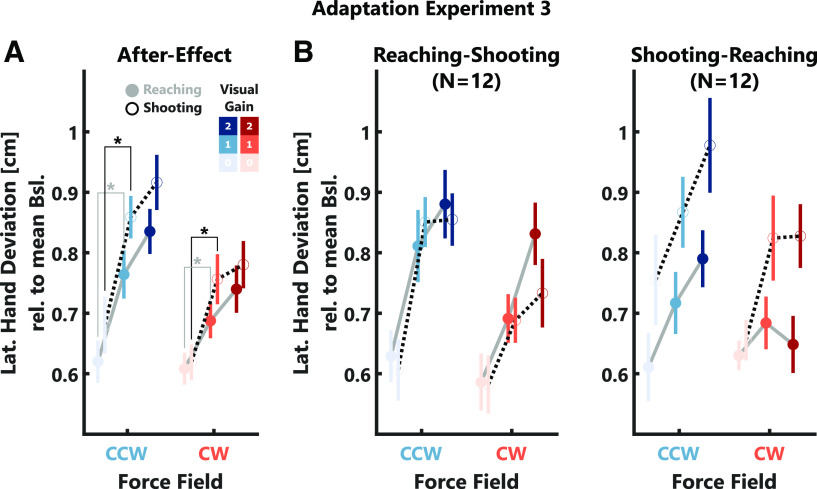
Adaptation data experiments 3. ***A***, Group average after-effects. Note, that values for CW perturbations were inverted to positive values for better illustration. The reaching task is represented by gray lines with filled-circles and the shooting task by black-dotted lines with open circles. ***B***, Average after-effects separated by task order. The left panel shows the data of participants that started with the reaching task, the right panel shows the data for the group that started with the shooting task. The color code is identical to Figure 2. Stars indicate significant differences determined using paired comparisons with *post hoc* Bonferroni corrections: *p < 0.05, **p < 0.001.

To investigate whether these differences in the use of visual feedback for online corrections during the reaching and shooting tasks influenced the use of vision for adaptation, we compared the after-effects between these two tasks (Fig. 8*A*). Similar to experiment 2, we observed a small increase in after-effect with increasing visual gain (CW: *t*_(3560)_ = 4.34, *p* < 10^−4^; CCW: *t*_(3553)_ = 6.12, *p* < 10^−4^; see Table 1, n) but no difference in adaptation depending on the task. *Post hoc* tests revealed that after-effects were significantly smaller for visual gain 0 compared with 1 in both tasks (*Reaching:* CW: *t*_(23)_ = −2.75, *p* = 0.034, Cohen’s *d*_av_ = −0.59; CCW: *t*_(23)_ = −3.31, *p* = 0.0091, Cohen’s *d*_av_ = −0.79; *Shooting:* CW: *t*_(23)_ = −3.15, *p* = 0.0135, Cohen’s *d*_av_ = −0.79; CCW: *t*_(23)_ = −4.14, *p* = 0.0012, Cohen’s *d*_av_ = −0.89), but there was no difference between visual gain 1 and 2. Importantly, we observed a similar increase of adaptation with the visual gain when we quantified the after-effect as the peak lateral force 50–150 ms after movement onset (CW: *t*_(3560)_ = 2.52, *p* = 0.0119; CCW: *t*_(3553)_ = 3.64, *p* = 0.0003; see Table 1, n) as well as no differences in after-effects between the two tasks. When we separated the after-effects depending on the order in which the tasks were performed, we observed similar after-effects across tasks for participants who started with the reaching task. However, participants who first performed the shooting task exhibited overall larger after-effects in the shooting task compared with the reaching task (Fig. 8*B*). For the same group, we observed larger maximum forward velocities in the shooting task. Therefore, stronger force perturbations might have resulted in overall larger adaptation during this task.

It is interesting that despite the large differences in feedback corrections between the two tasks, the adaptation responses were very similar. Thus, we performed an additional analysis to compare the relative contribution of hand errors and visual gain to the adaptation in the reaching and shooting task. Using bootstrapping to estimate the distributions of slope parameters, we observed a larger contribution of both maximum hand error and visual gain during the shooting task compared with the reaching task (maximum hand error: *t*_(999)_ = 26.79, *p* < 10^−4^; visual gain: *t*_(999)_ = −57.03, *p* < 10^−4^). This result suggests that the contribution of proprioceptive and visual errors was increased in the shooting task.

In summary, the findings of experiment 3 corroborate the results of experiments 1 and 2 regarding the contribution of visual feedback to online correction. Further, we observed that the contribution of visual feedback was almost eliminated when there was no need to correct the movement back to the target in the shooting task. Similar to experiment 2, we did observe a small increase in adaptive responses with increasing visual error in both tasks. Indeed, the adaptive responses were surprisingly similar across tasks, except that the contribution of hand and visual errors was larger in the shooting task. It is possible that visual errors influenced adaptation independent of feedback corrections because participants still paid attention to the visual feedback and associated it to their hand movement. Hence, in our last experiment we aimed to investigate whether explicitly informing participants about potential discrepancies between cursor and hand positions and instructing them to control their hidden hand instead of the cursor would reduce visual contributions to online control and adaptation.

### Experiment 4: instruction to control the cursor or the hand impacts visual contribution to online control and adaptation

In experiment 4, we explicitly instructed participants (*N* = 16) to either control their hidden hand or the visual cursor. Importantly, when they were asked to control their hand, we also informed them that the cursor might deviate from their true hand position during the experiment. Similar to experiment 3, all participants performed both instructions in sequence and the order was counterbalanced across participants. We were interested to see to which extend the differentiation between hand and cursor influenced the contributions of visual feedback to online control and adaptation.

**Figure 9.**
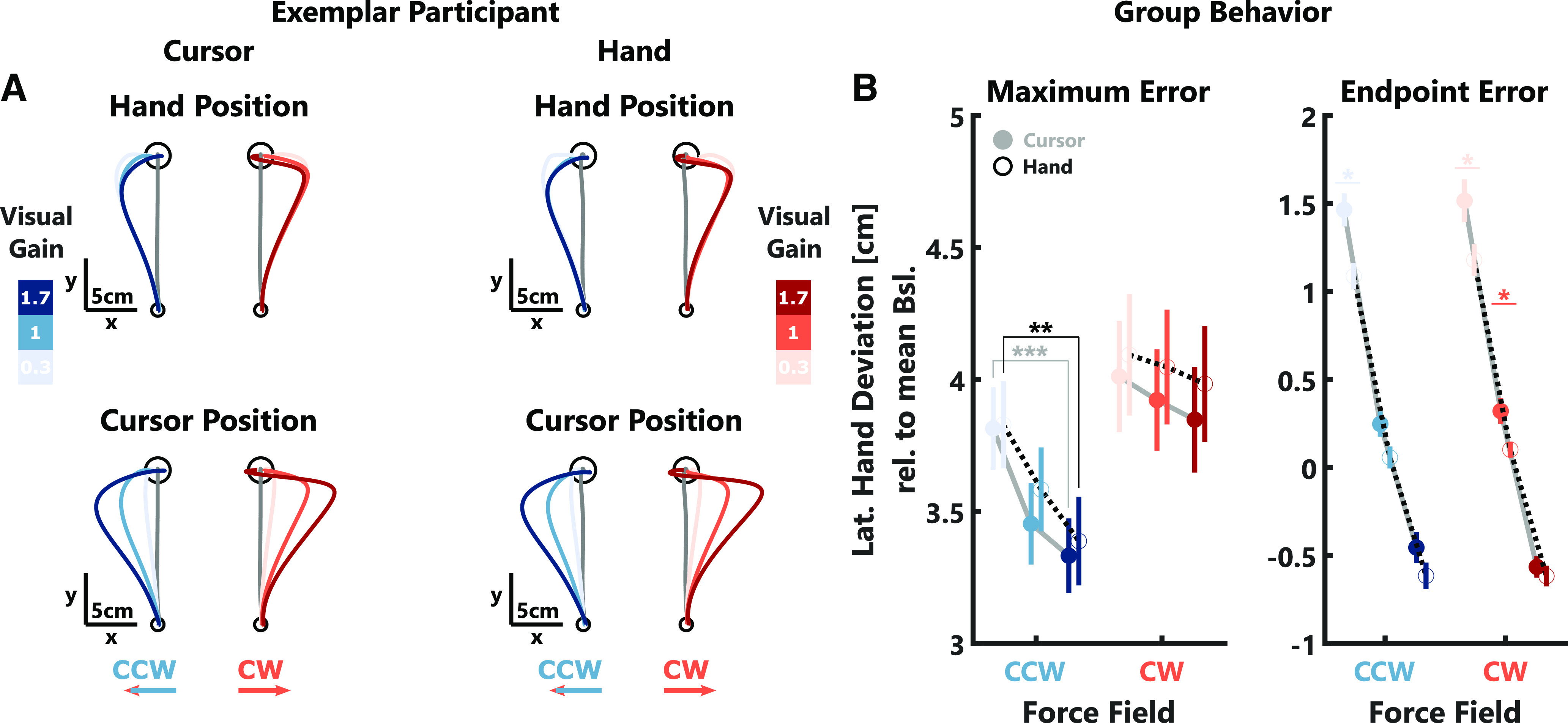
Behavioral data experiment 4. ***A***, Mean hand (top) and cursor (bottom) trajectories for one exemplar participant in the cursor-instruction (left) and hand-instruction task (right). ***B***, Group average maximum (left) and endpoint errors (right) ± SEM. Note, that values for CCW perturbations were inverted to positive values for better illustration. The cursor-instruction task is represented by gray lines and filled circles, the hand-instruction task by black-dotted lines and open circles. The color-code follows the explanation in Figure 2. Stars indicate significant differences determined using paired comparisons with *post hoc* Bonferroni corrections: *p < 0.05, **p < 0.001, ***p < 0.0001. See Extended Data Figure 9-1 for an illustration of the data separated by the order in which the tasks were performed.

As in previous experiments, the maximum errors significantly decreased with the visual gain (Fig. 9; CW: *t*_(2392)_ = −2.25, *p* = 0.0246; CCW: *t*_(2386)_ = −9.06, *p* < 10^−4^; see Table 1, o); however, there was no difference depending on the task instruction. Endpoint errors also exhibited a significant decrease with the visual gain (CW: *t*_(2392)_ = −36.22, *p* < 10^−4^; CCW: *t*_(2386)_ = −39.33, *p* < 10^−4^; see Table 1, p). In addition, endpoint errors were significantly smaller during the hand instruction task (CW: *t*_(2392)_ = −6.06, *p* < 10^−4^; CCW: *t*_(2386)_ = −6.98, *p* < 10^−4^; see Table 1, p) and decreased less strongly with the visual gain (CW: *t*_(2392)_ = 3.5, *p* = 0.0005; CCW: *t*_(2386)_ = 3.12, *p* = 0.0018; see Table 1, p). To control for potential effects of the order in which task instructions were performed, we again separated the data by task order. We observed similar patterns of maximum errors for both task order groups (Extended Data Fig. 9-1*A*). However, when comparing the endpoint errors across groups, it was visible that participants who started with the hand instruction task exhibited larger (more positive) endpoint errors during the cursor instruction task for trials with visual gain 1 and 1.7 compared with those that started with the cursor instruction task (Extended Data Fig. 9-1*B*).

10.1523/ENEURO.0068-23.2023.f9-1 Extended Data Figure 9-1 Experiment 4: effect of task order. ***A***, Group average maximum hand errors for experiment 4 separated by task-order. The left panel shows the data of participants that started with the cursor instruction task, the right panel shows the data for the group that started with the hand-instruction task. Note, that values for CCW perturbations were inverted to positive values for better illustration. The cursor-instruction task is represented by grey lines with filled-circles and the hand-instruction task by black-dotted lines with open circles. ***B***, Same as ***A*** but for the endpoint hand error. Note, that values for CCW perturbations were inverted to positive values for better illustration. The color-code is identical to Figure 2. Download Figure 9-1, EPS file.

**Figure 10.**
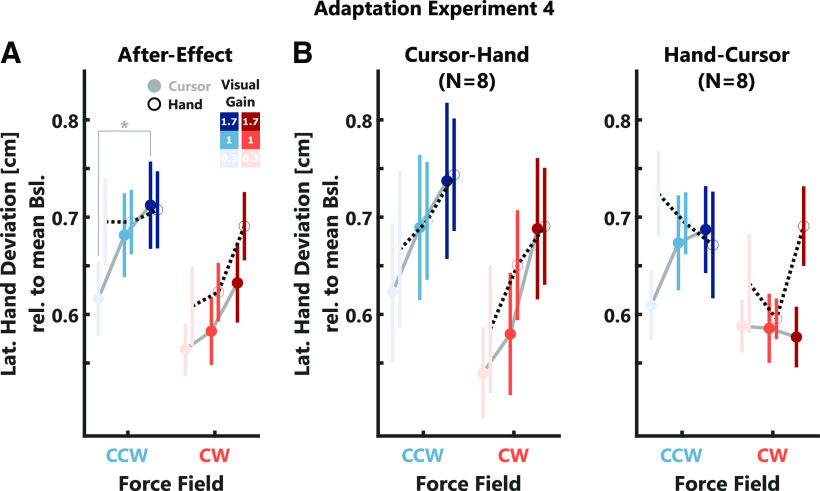
Adaptation data experiments 4. ***A***, Group average after-effects. Note, that values for CW perturbations were inverted to positive values for better illustration. The cursor-instruction task is represented by gray lines with filled-circles and the hand-instruction task by black-dotted lines with open circles. ***B***, Average after-effects separated by task order. The left panel shows the data of participants that started with the cursor-instruction task, the right panel shows the data for the group that started with the hand-instruction task. The color code is identical to Figure 2. Stars indicate significant differences determined using paired comparisons with *post hoc* Bonferroni corrections: *p < 0.05.

Similar to experiments 2 and 3, we observed a very small increase in after-effects with increasing visual gain, which was mostly present in CCW trials (CW: *t*_(2389)_ = 2.17, *p* = 0.0298; CCW: *t*_(2391)_ = 2.8, *p* = 0.0052; see Table 1, q). Additionally, during the CCW trials, after effects were slightly larger in the hand instruction compared with the cursor instruction task (CCW: *t*_(2391)_ = 2.24, *p* = 0.0255; see Table 1, q), but we did not observe a significant interaction between visual gain and task instruction (Fig. 10*A*). In a follow-up analysis, we quantified the after-effect as the peak lateral force 50–150 ms after movement onset and observed a similar increase in adaptation with increasing visual gain for the CW force-direction (CW: *t*_(2389)_ = 2.52, *p* = 0.0119; CCW: *t*_(2391)_ = 1.64, *p* > 0.05; see Table 1, q). Further, we again observed a slightly larger after-effect during CCW trials in the hand instruction task (CCW: *t*_(2391)_ = 2.15, *p* = 0.0317; see Table 1, q). Next, we separated the data for participants who began the experiment with the cursor instruction and those who began with the hand instruction task. Interestingly, we only observed a small but significant increase of after-effects with the visual gain across both tasks for the group that started with the cursor instruction, but not for those that started with the hand instruction (Fig. 10*B*; cursor-hand: CW: slope = 0.09, *t*_(1191)_ = 3.81, *p* = 0.0001, CI = [0.04,0.14]; CCW: slope = 0.07, *t*_(1195)_ = 2.65, *p* = 0.0082, CI = [0.02,0.12]; hand-cursor: CW: slope = 0.02, *t*_(1198)_ = 0.8, *p* > 0.05; CCW: slope = 0.01, *t*_(1196)_ = 0.39, *p* > 0.05). Similarly, when we quantified the after-effect as the early lateral force peak, we also obtained a significant effect of the visual gain in the group that started with the cursor instruction but not in the group that started with the hand instruction; however, this difference between task-orders was only present during CW trials (cursor-hand: CW: slope = 0.07, *t*_(1191)_ = 3.45, *p* = 0.0006, CI = [0.03,0.11]; CCW: slope = 0.04, *t*_(1195)_ = 1.58, *p* > 0.05; hand-cursor: CW: slope = 0.03, *t*_(1198)_ = 1.8, *p* > 0.05; CCW: slope = 0.02, *t*_(1196)_ = 0.83, *p* > 0.05).

The results of our final experiment demonstrate that the awareness about possible differences between hand and cursor position slightly reduced visually mediated corrections toward the end of the reach. Additionally, the small influence of the visual feedback on after-effects was eliminated for the group that was informed about potential divergences between the cursor and their hand at the beginning of the experiment. Hence, dissociating between hand and cursor reduced visual feedback contributions to online control and adaptation even if participants were not able to completely ignore the visual feedback during the perturbation.

### Relationship between limb errors and adaptation

**Figure 11.**
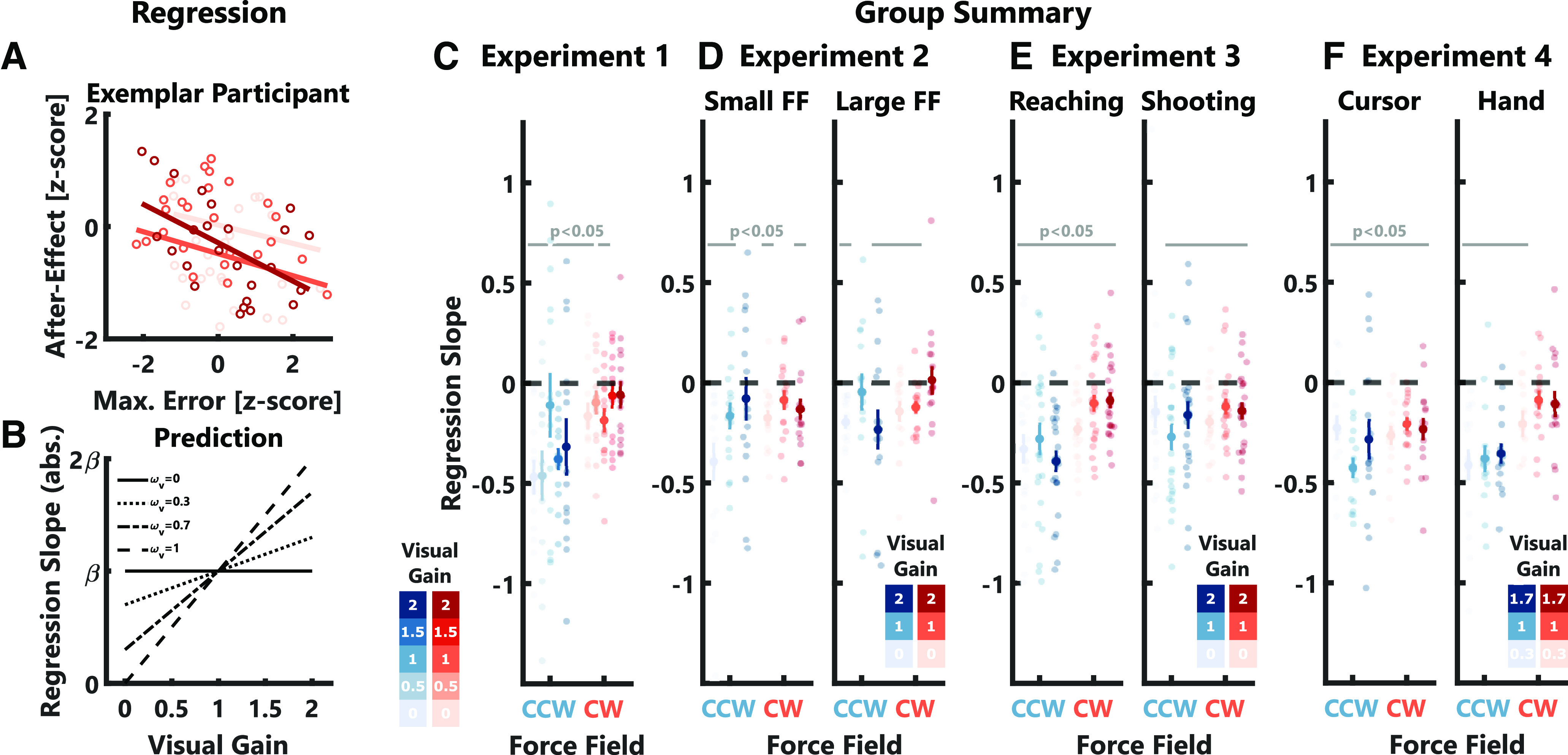
Relationship between maximum hand error and after-effect. ***A***, Relationship between the maximum hand error during perturbation and the after-effect on the subsequent trial for one exemplar participants in the shooting task of experiment 3 (only CW trials are shown). Regression slopes were computed for each visual gain condition separately and are shown in different shades of red. ***B***, Illustration of the predicted relationship between the visual gain and the regression slope, assuming a linear combination of visual and proprioceptive (limb afferent) estimates. Different possibilities for the size of the visual weight (ω_V_) are shown as full, dotted, and dashed lines. ***C***, Summary of regression slopes between maximum hand error and after effect computed for experiments 1. Full-colored markers indicate group average slopes ± SEM, transparent dots indicate single participant slope values. Horizontal gray lines indicate conditions for which the average slope was significant. The color-code is identical to Figure 2. ***D*–*F***, Same as ***C*** but for experiments 2–4.

Given that there was no consistent increase of the after-effect with the size of the visual error, we were interested to see in which way the felt hand deviation during each perturbation trial relates to the adaptation occurring on the very next trial. Thus, we looked at the relationship between the maximum hand deviation and the after-effect from individual trials. First, we *z*-transformed the maximum hand errors and after-effects relative to the mean of each participant in the visual gain 1 condition. Then, we computed the regression slopes separately for each participant and each value of the visual gain (Fig. 11*A*).

If we assume that visual and hand errors contribute to adaptation as a weighted combination, we can make a qualitative prediction regarding the relationship between hand errors and after-effects in the different visual gain conditions. Given this assumption, we should observe an increase in absolute slopes between after-effects and hand deviations with increasing visual gain (Fig. 11*B*). The reason is that visual gains larger than 1 should result in an overestimation of the hand error, which would ultimately lead to a larger update of the motor command for the next movement. Similarly, a visual gain lesser than 1 should result in an underestimation of the error and thus a smaller motor command update. The summary of each participants’ slope estimates in experiment 1 as well as the group averages can be seen in Figure 11*C*. We observed a statistically significant negative relationship between maximum hand errors and subsequent after-effects in six out of the ten conditions (two-tailed paired *t* test; −6.91 ≤ *t*_(13)_ ≤ −2.23, 10^−5^ < *p* < 0.05). Thus, the smaller the maximum hand deviation, the larger was the after-effect on the next trial. Comparable negative relationships between maximum hand errors and after-effects were observed in a majority of conditions across all experiments (Fig. 11*D–F*). In particular, in experiment 2 we observed significant negative slopes between the maximum error and the after-effects in four out of six conditions for the small force-field (−4.44 ≤ *t*_(15)_ ≤ −2.45, 0.0005 ≤ *p* ≤ 0.027) and in four out of six conditions for the large force-field (−5.61 ≤ *t*_(15)_ ≤ −2.33, 10^−4^ ≤ *p* ≤ 0.034). Similarly, in experiment 3, we observed on average a significant negative relationship in six out of six conditions for the reaching task (−7.22 ≤ *t*_(23)_ ≤ −2.15, 10^−4^ ≤ *p* ≤ 0.042) and in five out of six conditions in the shooting task (−5.97 ≤ *t*_(23)_ ≤ −2.29, 10^−4^ ≤ *p* ≤ 0.031). Lastly, in experiment 4, we found an average negative relationship between maximum error and after-effect in six out of six conditions in the cursor-instruction task (−8.27 ≤ *t*_(15)_ ≤ −2.78, 10^−4^ ≤ *p* ≤ 0.014) and in four out of six conditions in the hand-instruction task (−6.95 ≤ *t*_(15)_ ≤ −3.07, 10^−4^ ≤ *p* ≤ 0.008).

Given that the effect of the negative relationship between maximum error and after-effect was small and was not always significant on the individual participant level, we performed an additional analysis using linear-mixed models to verify the robustness of the group-level effect (Algermissen and Mehler, 2018). The models were fitted for each force-direction and visual gain combination separately. The results we obtained corroborated the findings reported in Figure 11. In experiment 1 we observed a significant negative relationship in six out of ten conditions (−6.11 ≤ *t*_(179–213)_ ≤ −2.04, 10^−4^ ≤ *p* ≤ 0.043). For experiment 2 we obtained significant results in two out of six conditions for small (−4.72 ≤ *t*_(371–375)_ ≤ −4.14, both *p* < 10^−4^) and large force-fields (−3.09 ≤ *t*_(374–377)_ ≤ −4.17, 10^−4^ ≤ *p* ≤ 0.002). In experiment 3 we observed a significant negative relationship between maximum error and after-effect in five out of six conditions for both the reaching task (−5.75 ≤ *t*_(577–589)_ ≤ −2.32, 10^−4^ ≤ *p* ≤ 0.02) and the shooting task (−3.9 ≤ *t*_(580–587)_ ≤ −2.15, 10^−4^ ≤ *p* ≤ 0.03). Lastly, in experiment 4 the effect was present in five out of six conditions in the cursor-instruction task (−5.48 ≤ *t*_(393–398)_ ≤ −2.26, 10^−4^ ≤ *p* ≤ 0.02) and in three out of six conditions in the hand-instruction task (−6.31 ≤ *t*_(394–397)_ ≤ −3.51, 10^−4^ ≤ *p* ≤ 0.0005).

Importantly, we did not observe a consistent relationship between the visual gain and the size of the regression slope across force-field directions and experiments. Hence, for a given size of the visual gain, stronger feedback corrections during the perturbation resulted in a stronger adaptation of the subsequent movement but this relationship was not modulated by the size of the visual error and did not differ between force-field strengths (experiment 2) or task instructions (experiments 3 and 4). Taken together, these observations suggest that the single trial after-effects in our experiment did not result from a weighted combination of visual and hand errors. Further, the absence of a systematic effect of vision on the regression slopes suggests that the visual error is not directly driving adaptation. Instead, the small impact of the visual error on the after-effect might be mediated indirectly by its modulation of the feedback response. Even in the shooting task of experiment 3, where feedback responses were minimized, the residual correction was sufficient to produce comparable fits with slope values that did not vary with the visual gain.

### Results summary

Across all four experiments, we observed a consistent contribution of visual feedback to online corrections. When participants had to counteract perturbations, their corrective responses scaled with the size of the visual error during small and large force-fields (experiments 1 and 2). This scaling was almost absent when the movement did not require corrections (experiment 3, shooting task) and was slightly reduced when participants were instructed to control their hidden hand instead of the visual cursor (experiment 4, hand instruction task). Importantly, the scaling of online corrections with the visual feedback did not systematically transfer to adaptive responses on the next trial. Nonetheless, our results demonstrate some influence of visual feedback on adaptation. First, while adaptation did not increase linearly with the size of the previously experienced visual error, after-effects were reduced following perturbation trials during which visual errors were eliminated (visual gain 0). Importantly, the elimination of visual errors even led to a suppression of the scaling of after-effects with the size of the perturbation force (experiment 2). Second, when participants were aware of potential divergences between the visual feedback and their felt hand location, they no longer exhibited a consistent visual influence on their adaptive responses (experiment 4). Lastly, we observed that stronger feedback corrections during perturbation resulted in larger adaptation of the next movement. However, this relationship was not influenced by the size of the visual error, which contradicts the assumption that motor adaptation is driven by a weighted combination of visual and hand errors.

## Discussion

Many previous studies have investigated the influence of different sensory error signals on motor adaptation using paradigms that minimize or eliminate feedback corrections during perturbation. We aimed to expand on these previous results by studying how the use of vision and limb afferent feedback for online control during perturbation modulates the contribution of these sensory signals to adapt the next movement. To this end, we increased or decreased visual errors relative to the true hand deviation in a random force-field paradigm. Our results show that online corrections during perturbation scaled with the size of the visual error but this scaling did not systematically translate into after-effects proportional to the visual error. Instead, we observed that for a given force-field magnitude and visual gain, after-effects decreased with increasing limb errors. Importantly, the slope of this negative relationship between limb error and adaptation did not show a consistent modulation with the visual gain, which is difficult to reconcile with a theory assuming linear combination of sensory signals. In summary, our results illustrate that feedback corrections and task instructions can modulate the contribution of visual error signals to adaptation.

We chose a random force-field adaptation paradigm to study the influence of visual and proprioceptive errors on online control and adaptation. The randomized perturbation schedule was necessary to ensure consistent feedback responses to perturbations throughout the length of the experiment and to be able to study their influence on single-trial adaptation. While such a random paradigm still allowed us to observe clear after-effects opposing the force-perturbation, error-based adaptation was unavoidably small as it resulted from the effect of a single perturbation trial. We quantified after-effects as the lateral deviation of the hand during null-field trials. Given that visual cursor feedback was present during these null-field trials, it is possible that this measure was also influenced by feedback corrections, which might have confounded the effect of the visual gain and the task instruction on the adaptation. However, after-effects exhibited a clear relationship with the force direction, force magnitude, and the applied feedback corrections, suggesting that they represent a reliable measure of the predictive component of adaptation. Further, in an alternative analysis we quantified the after-effect using the lateral forces applied early during null-field trials which are less likely influenced by feedback corrections. We observed a similar influence of the visual gain and the task instructions on adaptive responses using this alternative approach. Thus, while we cannot completely exclude the influence of feedback corrections on our measure of the after-effect, we are confident that it did not significantly impact our results. Randomizing force-direction and visual error size across trials resulted in a high level of unpredictability regarding the perturbations, which likely led participants to also employ a robust control strategy to reduce task errors (Crevecoeur et al., 2019). However, as shown by this recent study, it is likely that internal model-based and robust control strategies are active in parallel with the dominance in strategy depending on the level of unpredictability in the environment.

The results of experiment 1 differed from those of the other three experiments concerning the influence of the visual feedback on adaptation. One possibility why we did not observe an effect of vision on adaptation in experiment 1, is that we used a fully-randomized trial order. Consequently, there were fewer trials per condition and participants to extract after-effects and it is likely that encountering different perturbations in a row led to interactions that may have reduced differences in the size of the after-effects. Hence, in the subsequent experiments we chose a pseudo-randomized schedule during which each perturbation trial was followed by one null-field trial to measure the single-trial after-effect in a less random environment. Ideally, we could have increased the ratio between baseline and perturbation trials, but these experimental choices were constrained by the number of parameters and conditions that we explored. A detailed account of the influence of the perturbation schedule on behavior is an interesting question for future work.

The fact that we did not observe a systematic increase in after-effects with the size of the visual error contradicts the idea that adaptation followed a linear combination of visual and proprioceptive estimates, as suggested by models of optimal cue combination. This lack of scaling of the adaptive responses with the size of the visual error might be explained by previous studies showing that single-trial adaptive responses are similar despite differences in the preceding perturbation. For example, Fine and Thoroughman (2006) found that adaptive responses differed depending on the direction of a force-pulse, but they did not show specificity to the timing or magnitude of the perturbation. Similarly, Wei, et al. (2010) observed that single-trial adaptive responses showed a high correlation across various types of position-dependent force-fields. Our work builds up on these previous studies but we focused specifically on the influence of varying multisensory feedback on single-trial adaptation while the characteristics of the underlying perturbation remained the same. Importantly, contrary to these previous studies, we did observe a remaining influence of the visual error on adaptation. Specifically, while clamping the visual error to zero resulted in a reduction of after-effects, increasing the visual error relative to the actual limb displacement did not lead to a further increase in after-effects. Hence, our results suggest that there exists at least some degree of specificity of single-trial adaptive responses to the observed multisensory errors. Notably, the absence of a systematic scaling of the adaptive response with the visual error cannot be explained by a saturation of adaptation because after-effects clearly increased with the magnitude of the force-field in experiment 2. Therefore, additional factors need to be considered to explain the observed influence of visual and limb afferent feedback on adaptation. Our study clearly identifies two of these factors: a potentially prominent role of feedback correction to proprioceptive errors, which is itself modulated by visual errors, and the influence of task instructions.

The prevailing idea in the field of motor adaptation is that the nervous system gradually updates motor commands from one movement to the next based on the errors it observes during the movement (Smith et al., 2006; Kording et al., 2007). Some studies have proposed that the sensorimotor system directly uses corrective commands applied during perturbation to update the next movement (Kawato et al., 1987; Albert and Shadmehr, 2016). Therefore, an increase in feedback control should simultaneously result in smaller movement errors and larger after-effects. In line with this assumption, we observed a consistent negative relationship between the maximum hand errors during perturbation and the subsequent after-effect. Importantly, this relationship was not modulated by the size of the visual error, suggesting that the effect of visual feedback on adaptation in our experiments can be explained entirely by the influence it had on modulating feedback responses during perturbation. To our surprise, the negative relationship between limb errors and after-effects was also present in the shooting task of experiment 3, where participants were instructed not to correct their movements. It turns out that there remained residual corrections, potentially because of stretch responses and visuomotor reflexes, which participants were not able to suppress completely. Importantly, this observation questions our ability to probe adaptive responses in a putative feedforward controller as the feedback controller seems always present. A *post hoc* bootstrapping analysis revealed a larger contribution of maximum hand errors and visual gain to adaptation in the shooting compared with the reaching task, which suggests that, albeit small, there was indeed an influence of the task instruction on the contribution of sensory errors to adaptation. Additionally, we observed similar levels of adaptation to small and large force-fields in experiment 2 when the visual gain was zero and feedback corrections were reduced. These similarities in the size of the after-effect were observed despite large differences in movement errors between the two force-field magnitudes. Thus, future research on adaptation to multisensory movement errors should also consider how these errors are used for corrections during the movement.

Our findings regarding the asymmetric contribution of visual errors to adaptation corroborate several previous studies which have observed stagnations in adaptation with increasing error size. For instance, Marko and colleagues have argued that the sensitivity to error in the sensorimotor system decreases with error size resulting in a nonlinear relationship between the size of the error and the after-effect (Marko et al., 2012). More recently, Hayashi and colleagues have proposed that the pattern of visual and proprioceptive contributions to adaptation is best explained by a divisive normalization model (Hayashi et al., 2020). According to the authors, this model can explain why adaptation stagnates with error size even if visual and proprioceptive errors are congruent. Importantly, while we observed a stagnation in adaptation to increases in visual errors, there was a clear increase in adaptation with the force-magnitude. Hence, the magnitude of errors at which adaptation stagnates may differ across modalities, and might also depend on additional factors, such as the range of errors that are likely to be experienced during a specific motor task.

If adaptation would have relied on a weighted combination of visual and proprioceptive errors in our experiments, we should have observed an interaction between visual and limb errors on after-effects. The absence of such an interaction, suggests that visual and limb errors influenced motor adaptation in distinct ways. In fact, others have argued that vision and proprioception are not integrated during action, but rather that the selection of sensory feedback for movement control and adaptation can vary dramatically depending on the performed motor task. Several studies have reported a dominant reliance on visual feedback during adaptation of reaching movements when vision and proprioception provide contradictory input signals (Scheidt et al., 2005; Pipereit et al., 2006; Judkins and Scheidt, 2014). However, during dynamic perturbations of the limb when proprioception supplies important information to control and adapt movements, it can play a more prominent role compared with visual feedback (Bock and Thomas, 2011; Suminski et al., 2022). In accordance with these latter findings, our results highlight a clear dominance of force feedback over visual feedback during adaptation.

The results of experiment 4 also support the idea of task-dependent sensory contributions to control and adaptation. We observed that the influence of visual feedback on movement correction and adaptation was reduced when participants were instructed to control their hidden hand instead of the cursor. In line with the idea of causal inference, informing participants about potential discrepancies between the visual feedback and the hand, may have led them to discount visual information (Körding et al., 2007; Noppeney, 2021). An alternative explanation is that the instruction to control the hidden hand shifted participants attention more toward the limb afferent feedback which may have further reduced the influence of visual errors on adaptation. Previous studies have shown that attention can influences sensory weighting or even gate sensory signals used to make a perceptual judgment (Vercillo and Gori, 2015; Rohe and Noppeney, 2018). Similar to attention, reward or value associated with a specific modality stimulus has also been shown to influence multisensory weighting (Bean et al., 2021). In experiment 4, task success was determined either by the cursor or the hand location at the end of the movement, thus both attention and reward may have modulated the contribution of vision in this experiment. It is important to note that, even during the hand-instruction task, participants still relied on visual feedback to correct their movements during perturbation which demonstrates that they were unable to completely ignore the visual feedback. Further, we observed that the pattern of adaptation depended strongly on the first task instruction that was performed and that participants were not able to completely switch their behavior during the second task instruction. Nevertheless, our results provide additional support for a high degree of flexibility in how sensory stimuli are used and integrated to make decisions and control movements. Considering this task-dependent flexibility is integral to advancing our understanding of how the motor system uses multisensory error feedback.

Despite the lack of scaling of after-effects with the size of the visual error, we did observe a reduction in adaptation when visual movement errors were clamped to zero in experiments 2 and 3. Removing the visual hand deviation likely induced a noticeable mismatch between hand and cursor locations, which could have led to a segregation of hand and cursor feedback (Körding et al., 2007; Noppeney, 2021). However, contradictory to the observations of an earlier study (Scheidt et al., 2005), the clamping of visual movement errors did not completely override the influence of the limb afferent feedback in our experiments. This indicates that at least some degree of integration across sensory modalities was present in this condition. A previous study has shown that clamping visual feedback to a straight-line path increases the uncertainty of proprioceptive estimates of hand path curvature (Scheidt et al., 2010). Hence, an increase in proprioceptive uncertainty when visual errors were clamped to zero might have led participants to rely slightly more on visual error estimates in this condition leading to a small reduction in after-effects. Alternatively, removing the visual movement deviation could have directly influence the perceived lateral force leading to an illusion of a weaker force being applied to the hand. However, if this were the case, increasing the visual deviation should have led to an increase in the perceived force and thus an increase in adaptation. Future studies should systematically investigate the effect of the visual gain on the perception of applied forces and whether perceptual estimates show a similar asymmetric variation with the visual gain.

In summary, we observed a scaling of corrective responses with increasing visual errors. However, this scaling did not transfer one-to-one to after-effects, suggesting that visual feedback did not contribute in the same way to control and adaptation. Our findings corroborate previous studies indicating that our current theories of multisensory integration do not generalize to motor adaptation. Future research needs to consider additional behavioral variables, such as task instructions and the need for feedback corrections to account for the sensorimotor system’s flexibility in using and integrating sensory cues. Expanding current theories to include these variables will be integral to link models of feedback control and error-based adaptation.

## Synthesis

Reviewing Editor: David Franklin, Technische Universitat Munchen Decisions are customarily a result of the Reviewing Editor and the peer reviewers coming together and discussing their recommendations until a consensus is reached. When revisions are invited, a fact-based synthesis statement explaining their decision and outlining what is needed to prepare a revision will be listed below. The following reviewer(s) agreed to reveal their identity: Daichi Nozaki, Naotoshi Abekawa.

The reviewers were very happy with your revisions. There are only a couple of minor quick suggestions, which I will be very happy to accept when you resubmit.

Reviewer 1:

Thank you for your efforts in revising the manuscript. I am now satisfied with the revision.

Reviewer 2:

The authors have answered my concerns with additional analyses. In particular, the analysis assessing after-effects in the early response phase was quite useful in distinguishing between the effect of online correction and the effect of learning in null-field trials. They strengthen the main findings of the paper. I only have a few minor comments.

Minor

[1] Line 366: we observed visual gain-related changed ->: we observed visual gain-related changes

[2] Line 593: 4 out of six conditions -> four out of six conditions

[3] Regarding the binomial test of 6 out of 10, I originally thought the p should be 0.5 rather than 0.05, leading to insignificant effect (p=0.75). As the authors write in their rebuttal comment, using p=0.05 for a binomial test is, in my sense, a somewhat wild argument, in which case even a small number of participants having significant effects can be assessed as a broad trend. However, the authors have used a linear mixed model to examine global effects, which is convincing. I do not need any further responses on this statistical issue.

## Author Response

Answers to Reviewers

We sincerely thank the reviewers for their assessment of our work and we are pleased to read that our revisions have resolved all previous concerns. Below we added our replies to all remaining points of revision.

Please note that the indicated page and line references correspond to the latest revised version of our manuscript. Text references that refer to parts of the manuscript that were added or changed are highlighted in red. Similarly, text passages that we changed or added to the original manuscript are highlighted in red in our answers and in the text of the revised manuscript.

Synthesis of Reviews:

Synthesis Statement for Author (Required):

The reviewers were very happy with your revisions. There are only a couple of minor quick suggestions, which I will be very happy to accept when you resubmit.

Reviewer #1:

Thank you for your efforts in revising the manuscript. I am now satisfied with the revision.

Reviewer #2:

The authors have answered my concerns with additional analyses. In particular, the analysis assessing after-effects in the early response phase was quite useful in distinguishing between the effect of online correction and the effect of learning in null-field trials. They strengthen the main findings of the paper. I only have a few minor comments.

Minor

[1] Line 366: we observed visual gain-related changed ->: we observed visual gain-related changes

We corrected the spelling mistake on page 15, line 367 of the revised manuscript:

“Importantly, during the perturbation trials, we observed visual gain-related changes in EMG activity only after 150ms (see Fig. 3A), therefore these early force peaks are unlikely influenced by visual feedback corrections.”

[2] Line 593: 4 out of six conditions -> four out of six conditions

We made the requested change on page 24, line 594 of the revised manuscript:

“Lastly, in experiment 4, we found an average negative relationship between maximum error and after-effect in six out of six conditions in the cursor-instruction task (-8.27 {less than or equal to} t(15) {less than or equal to} -2.78, 10-4 {less than or equal to} p {less than or equal to} 0.014) and in four out of six conditions in the hand-instruction task (-6.95 {less than or equal to} t(15) {less than or equal to} -3.07, 10-4 {less than or equal to} p {less than or equal to} 0.008).”

For consistency we also spelled out all numbers in the following paragraph (pages 24-25, lines 596-609):

“Given that the effect of the negative relationship between maximum error and after-effect was small and was not always significant on the individual participant level, we performed an additional analysis using linear-mixed models to verify the robustness of the group-level effect (Algermissen and Mehler, 2018). The models were fitted for each force-direction and visual gain combination separately. The results we obtained corroborated the findings reported in figure 11. In experiment 1 we observed a significant negative relationship in six out of ten conditions (-6.11 {less than or equal to} t(179-213) {less than or equal to} -2.04, 10-4 {less than or equal to} p {less than or equal to} 0.043). For experiment 2 we obtained significant results in two out of six conditions for small (-4.72 {less than or equal to} t(371-375) {less than or equal to} -4.14, both p < 10-4) and large force-fields (-3.09 {less than or equal to} t(374-377) {less than or equal to} -4.17, 10-4 {less than or equal to} p {less than or equal to} 0.002). In experiment 3 we observed a significant negative relationship between maximum error and after-effect in five out of six conditions for both the reaching task (-5.75 {less than or equal to} t(577-589) {less than or equal to} -2.32, 10-4 {less than or equal to} p {less than or equal to} 0.02) and the shooting task (-3.9 {less than or equal to} t(580-587) {less than or equal to} -2.15, 10-4 {less than or equal to} p {less than or equal to} 0.03). Lastly, in experiment 4 the effect was present in five out of six conditions in the cursor-instruction task (-5.48 {less than or equal to} t(393-398) {less than or equal to} -2.26, 10-4 {less than or equal to} p {less than or equal to} 0.02) and in three out of six conditions in the hand-instruction task (-6.31 {less than or equal to} t(394-397) {less than or equal to} -3.51, 10-4 {less than or equal to} p {less than or equal to} 0.0005).”

[3] Regarding the binomial test of 6 out of 10, I originally thought the p should be 0.5 rather than 0.05, leading to insignificant effect (p=0.75). As the authors write in their rebuttal comment, using p=0.05 for a binomial test is, in my sense, a somewhat wild argument, in which case even a small number of participants having significant effects can be assessed as a broad trend. However, the authors have used a linear mixed model to examine global effects, which is convincing. I do not need any further responses on this statistical issue.

We thank the reviewer for the clarification of this point and we agree that the linear mixed model is more adequate to provide statistical support for the existence of a relationship between maximum hand errors and after-effects on the group level.
